# Supplementary material for: Hospital-treated infections in early- and mid-life and risk of Alzheimer’s disease, Parkinson’s disease, and amyotrophic lateral sclerosis: A nationwide nested case-control study in Sweden
Source: PLoS Med. 2022 Sep 15;19(9):e1004092. doi: 10.1371/journal.pmed.1004092 (PMC9477309; doi:10.1371/journal.pmed.1004092)
Supplement: S1 Table — (DOCX) [file pmed.1004092.s002.docx]

**Supplementary materials**

Hospital-treated infections in early- and mid-life and risk of Alzheimer’s disease, Parkinson’s disease, and amyotrophic lateral sclerosis: A nationwide nested case-control study in Sweden

Sun J, et al.

| S1 Table. The Swedish revisions of International Classification of Diseases (ICD) codes for neurodegenerative diseases | | | |
| --- | --- | --- | --- |
| Disease | ICD-8 (1969-1986) | ICD-9 (1987-1996) | ICD-10 (1997-) |
| Alzheimer's disease | 290 | 290A, 290B, 331A | F00, G30 |
| Parkinson's disease | 342 | 332A | G20 |
| Amyotrophic lateral sclerosis | 348,00 | 335C | G122 |
