# Supplementary material for: Hospital-treated infections in early- and mid-life and risk of Alzheimer’s disease, Parkinson’s disease, and amyotrophic lateral sclerosis: A nationwide nested case-control study in Sweden
Source: PLoS Med. 2022 Sep 15;19(9):e1004092. doi: 10.1371/journal.pmed.1004092 (PMC9477309; doi:10.1371/journal.pmed.1004092)
Supplement: S2 Table — (DOCX) [file pmed.1004092.s003.docx]

**Supplementary materials**

Hospital-treated infections in early- and mid-life and risk of Alzheimer’s disease, Parkinson’s disease, and amyotrophic lateral sclerosis: A nationwide nested case-control study in Sweden

Sun J, et al.

| S2 Table: The Swedish revisions of International Classification of Diseases (ICD) codes for hospital-treated infections | | | |
| --- | --- | --- | --- |
|  | ICD-8 | ICD-9 | ICD-10 |
| **Infection type** | | | |
| Bacterial | 000, 001, 002, 003, 004, 005, 073, 076, 080, 081, 082, 083, 320, 362, 380, 381, 382, 383, 421, 461, 481, 482, 501, 510, 567, 590, 595, 597, 612, 613, 614, 616, 620, 622, 630, 635, 670, 678, 680, 681, 682, 684, 710, 720, 010, 011, 012, 013, 014, 015, 016, 017, 018, 019, 020, 021, 022, 023, 024, 025, 026, 027, 030, 031, 032, 033, 034, 035, 036, 037, 038, 039, 090, 091, 092, 093, 094, 095, 096, 097, 098, 099, 100, 101, 102, 103, 104, 390, 391, 392 | 001, 002, 003, 004, 005, 073, 076, 077, 078, 079, 080, 081, 082, 083, 320, 381, 382, 383, 383, 421, 461, 475, 481, 482, 510, 567, 590, 595, 597, 670, 730, 010, 011, 012, 013, 014, 015, 016, 017, 018, 020, 021, 022, 023, 024, 025, 026, 027, 030, 031, 032, 033, 034, 035, 036, 037, 038, 039, 040, 041, 090, 091, 092, 093, 094, 095, 096, 097, 098, 099, 100, 101, 102, 103, 104, 390, 391, 392, 614, 615, 616, 680, 681, 682, 683, 684, 685, 686 | A00, A01, A02, A03, A04, A05, A15, A16, A17, A18, A19, A20, A21, A22, A23, A24, A25, A26, A27, A28, A30, A31, A32, A33, A34, A35, A36, A37, A38, A39, A40, A41, A42, A43, A44, A45, A46, A47, A48, A49, A50, A51, A52, A53, A54, A55, A56, A57, A58, A65, A66, A67, A68, A69, A70, A71, A72, A73, A74, A75, A76, A77, A78, A79, B95, B96, G00, G01, H60, H70, I00, I01, I02, I33, J01, J13, J14, J15, J36, J86, K65, L00, L01, L02, L03, L04, L05, L06, L07, L08, M00, M86, N30, N34, N70, N71, N72, N73, N74, N75, N76, N77, O23, O85, O86, P36 |
| Viral | 075, 360, 420, 422, 460, 464, 465, 466, 480, 040, 041, 042, 043, 044, 045, 046, 050, 051, 052, 053, 054, 055, 056, 057, 060, 061, 062, 063, 064, 065, 066, 067, 068, 070, 071, 072, 073, 074, 075, 076, 077, 078, 079, 470, 471, 472, 473, 474 | 070, 071, 072, 074, 075, 077, 078, 079, 372, 420, 422, 460, 464, 465, 466, 480, 487, 647, 711, 045, 046, 047, 048, 049, 050, 051, 052, 053, 054, 055, 056, 057, 060, 061, 062, 063, 064, 065, 066 | A08, A60, A80, A81, A82, A83, A84, A85, A86, A87, A88, A89, A90, A91, A92, A93, A94, A95, A96, A97, A98, A99, B00, B01, B02, B03, B04, B05, B06, B07, B08, B09, B15, B16, B17, B18, B19, B20, B21, B22, B23, B24, B25, B26, B27, B28, B29, B30, B31, B32, B33, B34, B97, B99, H10, I30, I40, J00, J04, J05, J06, J10, J12, J20, J21, O98, P35, Z21 |
| Others | 084, 085, 086, 087, 088, 089, 112, 113, 114, 117, 120, 121, 122, 124, 125, 126, 128, 130, 131, 132, 133, 134, 135, 136, 363, 610, 611, 615, 732, 763, Y41 | 084, 085, 086, 087, 088, 112, 113, 114, 117, 118, 120, 121, 122, 124, 125, 126, 128, 130, 131, 132, 133, 134, 135, 136, 370, 675, 771, 137, 138, 139 | V02, B37, B38, B45, B46, B47, B48, B49, B50, B51, B52, B53, B54, B55, B56, B57, B58, B60, B61, B62, B63, B64, B65, B66, B67, B68, B69, B70, B72, B73, B74, B75, B76, B77, B78, B79, B80, B83, B85, B86, B87 B88, B89, B90, B91, B92, B93, B94, H16, H32, M01, M02, M03, O91, P37, P38, P39, Z22, A59, A63, A64 |
| **Infection site** | | | |
| CNS | 013, 062, 063, 064, 065, 066, 071, 094, 292, 320, 323, 324, 390, 474, 040, 041, 042, 043, 044, 045, 046 | 013, 062, 063, 064, 071, 094, 320, 323, 326, 392, 045, 046, 047, 048, 049 | A17, A80, A81, A82, A83, A84, A85, A86, A87, A88, A89, G00, G01, G02, G04, G05, I02 |
| Gastrointestinal | 014, 123, 127, 129, 540, 567, 000, 001, 002, 003, 004, 005, 006, 007, 008, 009 | 014, 123, 127, 129, 540, 567, 000, 001, 002, 003, 004, 005, 006, 007, 008, 009 | A00, A01, A02, A03, A04, A05, A06, A07, A08, A09, B71, B81, B82, K35, K65, K67 |
| Genitourinary | 016, 590, 595, 597 | 016, 590, 595, 597 | N30, N34, O23, N390 |
| Respiratory | 010, 011, 012, 033, 034, 075, 115, 116, 490, 501, 503, 510, 460, 461, 462, 463, 464, 465, 466, 470, 471, 472, 473, 474, 480, 481, 482, 483, 484, 485, 486 | 010, 011, 012, 033, 034, 075, 115, 116, 473, 475, 487, 490, 510, 460, 461, 462, 463, 464, 465, 466, 480, 481, 482, 483, 484, 485, 486 | A15, A16, A37, A38, B27, B39, B40, B41, B42, B44, B59, J00, J01, J02, J03, J04, J05, J06, J10, J12, J13, J14, J15, J16, J17, J18, J20, J21, J22, J32, J36, J40, J41, J42, J86, P23 |
| Skin | 110, 111, 050, 051, 052, 053, 054, 055, 056, 057, 680, 681, 682, 683, 684, 685, 686 | 110, 111, 050, 051, 052, 053, 054, 055, 056, 057, 680, 681, 682, 683, 684, 685, 686 | B00, B01, B02, B03, B04, B05, B06, B07, B08, B09, B35, B36, B43, L00, L01, L02, L03, L04, L05, L06, L07, L08 |
