# Supplementary material for: Hospital-treated infections in early- and mid-life and risk of Alzheimer’s disease, Parkinson’s disease, and amyotrophic lateral sclerosis: A nationwide nested case-control study in Sweden
Source: PLoS Med. 2022 Sep 15;19(9):e1004092. doi: 10.1371/journal.pmed.1004092 (PMC9477309; doi:10.1371/journal.pmed.1004092)
Supplement: S4 Table — (DOCX) [file pmed.1004092.s005.docx]

**Supplementary materials**

Hospital-treated infections in early- and mid-life and risk of Alzheimer’s disease, Parkinson’s disease, and amyotrophic lateral sclerosis: A nationwide nested case-control study in Sweden

Sun J, et al.

| S4 Table. Association between any hospital-treated infection and risk of neurodegenerative disease, stratified analyses by sex, age at diagnosis, calendar period at diagnosis, and year of birth (5-year lag time) | | | | | | | | | | | | | | |
| --- | --- | --- | --- | --- | --- | --- | --- | --- | --- | --- | --- | --- | --- | --- |
|  | AD | | | |  | PD | | | |  | ALS | | | |
| Group | Infection (case/control) | No infection (case/control) | OR (95% CI) | *P* |  | Infection (case/control) | No infection (case/control) | OR (95% CI) | *P* |  | Infection (case/control) | No infection (case/control) | OR (95% CI) | *P* |
| Sex |  |  |  |  |  |  |  |  |  |  |  |  |  |  |
| Male | 20908/92009 | 115090/587981 | 1.20 (1.18-1.22) | <0.001 |  | 8748/44067 | 48533/242338 | 1.01 (0.98-1.03) | 0.536 |  | 903/4700 | 4870/24165 | 0.95 (0.88-1.04) | 0.260 |
| Female | 28881/133767 | 127062/645948 | 1.13 (1.12-1.15) | <0.001 |  | 8070/37824 | 38568/195366 | 1.07 (1.05-1.11) | <0.001 |  | 890/4484 | 3498/17456 | 1.00 (0.92-1.09) | 0.960 |
| Age at diagnosis |  |  |  |  |  |  |  |  |  |  |  |  |  |  |
| < 60 y | 13196/37418 | 62827/342697 | 1.93 (1.89-1.98) | <0.001 |  | 2110/8600 | 11209/57995 | 1.29 (1.22-1.36) | <0.001 |  | 430/2125 | 1815/9100 | 1.02 (0.90-1.16) | 0.750 |
| ≥ 60 y | 36593/188358 | 179325/891232 | 1.00 (0.98-1.01) | 0.508 |  | 14708/73291 | 75892/379709 | 1.01 (0.99-1.03) | 0.382 |  | 1363/7059 | 6553/32521 | 0.96 (0.90-1.03) | 0.243 |
| Calendar period at diagnosis |  |  |  |  |  |  |  |  |  |  |  |  |  |  |
| 1970-1986 | 4770/20532 | 65062/328628 | 1.32 (1.27-1.36) | <0.001 |  | 1674/7894 | 27418/137566 | 1.14 (1.08-1.20) | <0.001 |  | 95/567 | 2128/10548 | 0.85 (0.67-1.06) | 0.146 |
| 1987-2000 | 23945/98429 | 120947/626031 | 1.27 (1.25-1.29) | <0.001 |  | 4832/22506 | 28961/146459 | 1.06 (1.02-1.09) | 0.002 |  | 306/1615 | 2188/10855 | 0.93 (0.81-1.06) | 0.263 |
| 2001-2016 | 21074/106815 | 56143/279270 | 0.98 (0.97-1.00) | 0.063 |  | 10312/51491 | 30722/153679 | 1.00 (0.98-1.03) | 0.750 |  | 1392/7002 | 4052/20218 | 1.00 (0.94-1.08) | 0.893 |
| Year of birth |  |  |  |  |  |  |  |  |  |  |  |  |  |  |
| 1900-1919 | 15858/84379 | 118439/587106 | 0.97 (0.96-0.99) | 0.004 |  | 4837/23902 | 42993/215248 | 1.02 (0.99-1.06) | 0.236 |  | 221/1208 | 2590/12847 | 0.91 (0.78-1.06) | 0.239 |
| 1920-1939 | 19762/96900 | 68988/346850 | 1.05 (1.03-1.06) | <0.001 |  | 7774/39022 | 31175/155723 | 1.00 (0.97-1.03) | 0.941 |  | 737/3973 | 3509/17257 | 0.91 (0.83-0.99) | 0.032 |
| 1940-1959 | 9387/29213 | 36470/200072 | 1.78 (1.73-1.84) | <0.001 |  | 3572/16637 | 10649/54468 | 1.08 (1.04-1.13) | <0.001 |  | 674/3286 | 1965/9909 | 1.07 (0.97-1.18) | 0.196 |
| ≥ 1960 | 4782/15284 | 18255/99901 | 1.59 (1.52-1.65) | <0.001 |  | 635/2330 | 2284/12265 | 1.39 (1.24-1.56) | <0.001 |  | 161/717 | 304/1608 | 1.11 (0.88-1.40) | 0.366 |
| AD: Alzheimer’s disease; ALS: amyotrophic lateral sclerosis; CI, confidence interval; OR, odds ratio; PD: Parkinson’s disease. Conditional on matching factors (sex and year of birth) and further adjusted for area of residence, educational attainment, family history of the disease, and history of comorbidity. Infections diagnosed during five years before the index date were excluded to alleviate the potential influence of reverse causation due to diagnostic delay and surveillance bias. | | | | | | | | | | | | | | |
