# Supplementary material for: Hospital-treated infections in early- and mid-life and risk of Alzheimer’s disease, Parkinson’s disease, and amyotrophic lateral sclerosis: A nationwide nested case-control study in Sweden
Source: PLoS Med. 2022 Sep 15;19(9):e1004092. doi: 10.1371/journal.pmed.1004092 (PMC9477309; doi:10.1371/journal.pmed.1004092)
Supplement: S5 Table — (DOCX) [file pmed.1004092.s006.docx]

**Supplementary materials**

Hospital-treated infections in early- and mid-life and risk of Alzheimer’s disease, Parkinson’s disease, and amyotrophic lateral sclerosis: A nationwide nested case-control study in Sweden

Sun J, et al.

| S5 Table. Associations between age at hospital-treated infection and the consequent risks of early-onset AD and late-onset AD (5-year lag time) | | | | | | | | | |
| --- | --- | --- | --- | --- | --- | --- | --- | --- | --- |
|  | Early onset AD | | | |  | Late onset AD | | | |
| Group | Infection (case/control) | No infection (case/control) | OR (95% CI) | *P* |  | Infection (case/control) | No infection (case/control) | OR (95% CI) | *P* |
| Age at infection (yes/no) |  |  |  |  |  |  |  |  |  |
| <40 y | 12178/34649 | 73525/393866 | 1.95 (1.91-2.00) | <0.001 |  | 1493/6885 | 204745/1024305 | 1.11 (1.05-1.18) | <0.001 |
| 40-59.9 y | 2811/9734 | 82892/418781 | 1.47 (1.40-1.54) | <0.001 |  | 9622/46685 | 196616/984505 | 1.07 (1.05-1.10) | <0.001 |
| ≥ 60 y | 0/9 | 85703/428506 | - | - |  | 27014/143464 | 179224/887726 | 0.96 (0.94-0.97) | <0.001 |
| Age at infection (frequency) |  |  |  |  |  |  |  |  |  |
| <40 y |  |  |  |  |  |  |  |  |  |
| 0 | - | 73525/393866 | Ref. |  |  | - | 204745/1024305 | Ref. |  |
| 1 | 8366/26863 | - | 1.74 (1.70-1.79) | <0.001 |  | 1241/5793 | - | 1.10 (1.03-1.17) | 0.003 |
| ≥ 2 | 3812/7786 | - | 2.70 (2.59-2.81) | <0.001 |  | 252/1092 | - | 1.19 (1.03-1.37) | 0.016 |
| 40-59.9 y |  |  |  |  |  |  |  |  |  |
| 0 | - | 82892/418781 | Ref. |  |  | - | 196616/984505 | Ref. |  |
| 1 | 1982/7142 | - | 1.41 (1.34-1.49) | <0.001 |  | 7521/36161 | - | 1.08 (1.05-1.11) | <0.001 |
| ≥ 2 | 829/2592 | - | 1.63 (1.50-1.77) | <0.001 |  | 2101/10524 | - | 1.05 (1.00-1.10) | 0.065 |
| ≥ 60 y |  |  |  |  |  |  |  |  |  |
| 0 | - | - | - | - |  | - | 179224/887726 | Ref. |  |
| 1 | - | - | - | - |  | 18520/96092 | - | 0.98 (0.96-0.99) | 0.004 |
| ≥ 2 | - | - | - | - |  | 8494/47372 | - | 0.92 (0.90-0.94) | <0.001 |
| AD: Alzheimer’s disease; CI, confidence interval; OR, odds ratio. The cut-off between early onset and late onset AD is 65 years old. Conditional on matching factors (sex and year of birth) and further adjusted for area of residence, educational attainment, family history of the disease, and history of comorbidity. Infections diagnosed during five years before the index date were excluded to alleviate the potential influence of reverse causation due to diagnostic delay. | | | | | | | | | |
