# Supplementary material for: Hospital-treated infections in early- and mid-life and risk of Alzheimer’s disease, Parkinson’s disease, and amyotrophic lateral sclerosis: A nationwide nested case-control study in Sweden
Source: PLoS Med. 2022 Sep 15;19(9):e1004092. doi: 10.1371/journal.pmed.1004092 (PMC9477309; doi:10.1371/journal.pmed.1004092)
Supplement: S6 Table — (DOCX) [file pmed.1004092.s007.docx]

**Supplementary materials**

Hospital-treated infections in early- and mid-life and risk of Alzheimer’s disease, Parkinson’s disease, and amyotrophic lateral sclerosis: A nationwide nested case-control study in Sweden

Sun J, et al.

| S6 Table. Association between hospital-treated infection and risk of neurodegenerative disease diagnosed at 60 years or older | | | | | | | | |
| --- | --- | --- | --- | --- | --- | --- | --- | --- |
| Group | AD | |  | PD | |  | ALS | |
|  | OR (95% CI) | *P* |  | OR (95% CI) | *P* |  | OR (95% CI) | *P* |
| Analysis by characteristics of infections | | | | | | | | |
| Infection type |  |  |  |  |  |  |  |  |
| Bacterial | 0.99 (0.97-1.00) | 0.155 |  | 1.01 (0.99-1.04) | 0.340 |  | 0.98 (0.90-1.06) | 0.564 |
| Viral | 0.97 (0.95-0.99) | 0.005 |  | 1.03 (1.00-1.07) | 0.078 |  | 1.06 (0.95-1.18) | 0.320 |
| Others | 1.02 (0.99-1.06) | 0.263 |  | 1.04 (0.98-1.10) | 0.159 |  | 1.12 (0.96-1.31) | 0.143 |
| Infection site |  |  |  |  |  |  |  |  |
| CNS | 0.95 (0.89-1.01) | 0.099 |  | 1.01 (0.92-1.11) | 0.846 |  | 0.74 (0.53-1.04) | 0.080 |
| Gastrointestinal | 1.11 (1.08-1.14) | <0.001 |  | 1.04 (1.00-1.09) | 0.077 |  | 0.92 (0.80-1.07) | 0.286 |
| Respiratory | 0.94 (0.92-0.96) | <0.001 |  | 0.96 (0.93-0.99) | 0.004 |  | 1.05 (0.95-1.16) | 0.338 |
| Genitourinary | 1.02 (0.99-1.05) | 0.188 |  | 1.14 (1.08-1.20) | <0.001 |  | 0.97 (0.81-1.18) | 0.783 |
| Skin | 0.99 (0.95-1.03) | 0.651 |  | 0.99 (0.93-1.05) | 0.644 |  | 0.92 (0.76-1.13) | 0.429 |
| Age at infection |  |  |  |  |  |  |  |  |
| <40 y | 1.12 (1.07-1.19) | <0.001 |  | 1.04 (0.98-1.11) | 0.165 |  | 1.02 (0.88-1.19) | 0.778 |
| 40-59.9 y | 1.09 (1.07-1.11) | <0.001 |  | 1.03 (1.00-1.06) | 0.061 |  | 1.03 (0.94-1.12) | 0.572 |
| ≥ 60 y | 0.96 (0.94-0.97) | <0.001 |  | 0.99 (0.97-1.02) | 0.604 |  | 0.89 (0.82-0.98) | 0.017 |
| Age and frequency of infection |  |  |  |  |  |  |  |  |
| <40 y |  |  |  |  |  |  |  |  |
| 0 | Ref. |  |  | Ref. |  |  | Ref. |  |
| 1 | 1.11 (1.05-1.18) | <0.001 |  | 1.03 (0.97-1.11) | 0.345 |  | 1.03 (0.87-1.21) | 0.747 |
| ≥ 2 | 1.18 (1.04-1.33) | 0.009 |  | 1.10 (0.96-1.25) | 0.187 |  | 1.00 (0.70-1.41) | 0.984 |
| 40-59.9 y |  |  |  |  |  |  |  |  |
| 0 | Ref. |  |  | Ref. |  |  | Ref. |  |
| 1 | 1.09 (1.06-1.12) | <0.001 |  | 1.02 (0.99-1.06) | 0.214 |  | 1.01 (0.91-1.12) | 0.827 |
| ≥ 2 | 1.08 (1.04-1.13) | <0.001 |  | 1.05 (0.99-1.12) | 0.087 |  | 1.07 (0.91-1.26) | 0.433 |
| ≥ 60 y |  |  |  |  |  |  |  |  |
| 0 | Ref. |  |  | Ref. |  |  | Ref. |  |
| 1 | 0.98 (0.96-0.99) | 0.006 |  | 1.00 (0.97-1.03) | 0.907 |  | 0.89 (0.79-0.99) | 0.027 |
| ≥ 2 | 0.92 (0.90-0.94) | <0.001 |  | 0.98 (0.94-1.02) | 0.418 |  | 0.91 (0.78-1.07) | 0.257 |
| Stratified analysis by sex, calendar period, and birth cohort | | | | | | | | |
| Sex |  |  |  |  |  |  |  |  |
| Male | 1.00 (0.98-1.02) | 0.928 |  | 0.99 (0.96-1.02) | 0.402 |  | 0.92 (0.84-1.02) | 0.105 |
| Female | 0.99 (0.98-1.01) | 0.356 |  | 1.03 (1.00-1.07) | 0.024 |  | 1.00 (0.91-1.10) | 0.995 |
| Calendar period at diagnosis |  |  |  |  |  |  |  |  |
| 1970-1986 | 0.94 (0.90-0.98) | 0.003 |  | 1.09 (1.02-1.15) | 0.008 |  | 0.74 (0.57-0.97) | 0.031 |
| 1987-2000 | 1.01 (0.99-1.03) | 0.433 |  | 0.99 (0.95-1.03) | 0.549 |  | 0.95 (0.82-1.11) | 0.536 |
| 2001-2016 | 0.98 (0.96-0.99) | 0.007 |  | 1.00 (0.97-1.03) | 0.992 |  | 0.99 (0.91-1.07) | 0.780 |
| Year of birth |  |  |  |  |  |  |  |  |
| 1900-1919 | 0.97 (0.95-0.99) | 0.002 |  | 1.02 (0.98-1.05) | 0.325 |  | 0.90 (0.77-1.05) | 0.183 |
| 1920-1939 | 1.00 (0.98-1.02) | 0.800 |  | 0.99 (0.96-1.02) | 0.414 |  | 0.91 (0.83-1.00) | 0.040 |
| 1940-1959 | 1.09 (1.03-1.15) | 0.002 |  | 1.06 (1.01-1.12) | 0.032 |  | 1.12 (0.99-1.27) | 0.071 |
| AD: Alzheimer’s disease; ALS: amyotrophic lateral sclerosis; CI, confidence interval; CNS: the central nervous system; OR, odds ratio; PD: Parkinson’s disease. Conditional on matching factors (age and sex) and further adjusted for area of residence, educational attainment, family history of the disease, and history of comorbidity. Infections diagnosed during five years before the index date were excluded to alleviate the potential influence of reverse causation due to diagnostic delay. | | | | | | | | |
