# Supplementary material for: Hospital-treated infections in early- and mid-life and risk of Alzheimer’s disease, Parkinson’s disease, and amyotrophic lateral sclerosis: A nationwide nested case-control study in Sweden
Source: PLoS Med. 2022 Sep 15;19(9):e1004092. doi: 10.1371/journal.pmed.1004092 (PMC9477309; doi:10.1371/journal.pmed.1004092)
Supplement: S7 Table — (DOCX) [file pmed.1004092.s008.docx]

**Supplementary materials**

Hospital-treated infections in early- and mid-life and risk of Alzheimer’s disease, Parkinson’s disease, and amyotrophic lateral sclerosis: A nationwide nested case-control study in Sweden

Sun J, et al.

| S7 Table. Sensitivity analyses for the association between any hospital-treated infection and risk of neurodegenerative disease (5-year lag time) | | | | | | | | | | | | | | |
| --- | --- | --- | --- | --- | --- | --- | --- | --- | --- | --- | --- | --- | --- | --- |
|  | AD | | | |  | PD | | | |  | ALS | | | |
| Sensitivity analysis | Infection (case/control) | No infection (case/control) | OR (95% CI) | *P* |  | Infection (case/control) | No infection (case/control) | OR (95% CI) | *P* |  | Infection (case/control) | No infection (case/control) | OR (95% CI) | *P* |
| Without a family history of the disease | 45549/217164 | 228047/1188019 | 1.13 (1.12-1.15) | <0.001 |  | 16240/80235 | 85302/432272 | 1.04 (1.02-1.06) | <0.001 |  | 1748/9159 | 8232/41520 | 0.97 (0.91-1.03) | 0.312 |
| At least two hospital visits about the same disease | 26629/124081 | 126908/643604 | 1.13 (1.11-1.15) | <0.001 |  | 11407/58676 | 58736/292039 | 1.03 (1.02-1.06) | <0.001 |  | 1398/7208 | 6087/30217 | 0.97 (0.91-1.04) | 0.354 |
| In whole population, without adjust for education | 49789/225776 | 242152/1233929 | 1.16 (1.15-1.18) | <0.001 |  | 16818/81891 | 87101/437704 | 1.04 (1.02-1.06) | <0.001 |  | 1793/9184 | 8368/41621 | 0.97 (0.92-1.03) | 0.379 |
| In those with data available for education, adjust for education | 32598/151528 | 107303/547548 | 1.103 (1.087-1.118) | <0.001 |  | 12849/63690 | 45211/225638 | 1.034 (1.012-1.054) | 0.012 |  | 1602/8096 | 5438/26935 | 0.984 (0.923-1.049) | 0.613 |
| In those with data available for education, not adjust for education | 32598/151528 | 107303/547548 | 1.103 (1.088-1.119) | <0.001 |  | 12849/63690 | 45211/225638 | 1.033 (1.011-1.052) | 0.017 |  | 1602/8096 | 5438/26935 | 0.983 (0.922-1.048) | 0.602 |
| After excluding individuals with multiple neurodegenerative diseases | 47802/216421 | 229712/1171149 | 1.18 (1.16-1.19) | <0.001 |  | 15273/74692 | 76595/384648 | 1.04 (1.02-1.06) | <0.001 |  | 1696/8801 | 8020/39779 | 0.97 (0.91-1.03) | 0.323 |
| AD: Alzheimer’s disease; ALS: amyotrophic lateral sclerosis; CI, confidence interval; OR, odds ratio; PD: Parkinson’s disease. Conditional on matching factors (sex and year of birth) and further adjusted for area of residence, educational attainment, family history of the disease, and history of comorbidity. Infections diagnosed during five years before the index date were excluded to alleviate the potential influence of reverse causation due to diagnostic delay and surveillance bias. | | | | | | | | | | | | | | |
