# Supplementary material for: Hospital-treated infections in early- and mid-life and risk of Alzheimer’s disease, Parkinson’s disease, and amyotrophic lateral sclerosis: A nationwide nested case-control study in Sweden
Source: PLoS Med. 2022 Sep 15;19(9):e1004092. doi: 10.1371/journal.pmed.1004092 (PMC9477309; doi:10.1371/journal.pmed.1004092)
Supplement: S9 Table — (DOCX) [file pmed.1004092.s010.docx]

**Supplementary materials**

Hospital-treated infections in early- and mid-life and risk of Alzheimer’s disease, Parkinson’s disease, and amyotrophic lateral sclerosis: A nationwide nested case-control study in Sweden

Sun J, et al.

| S9 Table. Associations between age at hospital-treated infection and the consequent risks of neurodegenerative diseases (10-year lag time) | | | | | | | | | | | | | | |
| --- | --- | --- | --- | --- | --- | --- | --- | --- | --- | --- | --- | --- | --- | --- |
|  | AD | | | |  | PD | | | |  | ALS | | | |
| Group | Infection (case/control) | No infection (case/control) | OR (95% CI) | *P* |  | Infection (case/control) | No infection (case/control) | OR (95% CI) | *P* |  | Infection (case/control) | No infection (case/control) | OR (95% CI) | *P* |
| Age at infection (yes/no) |  |  |  |  |  |  |  |  |  |  |  |  |  |  |
| <40 y | 10011/32135 | 271185/1373845 | 1.69 (1.65-1.73) | <0.001 |  | 2565/11637 | 97097/486673 | 1.12 (1.07-1.18) | <0.001 |  | 512/2584 | 9289/46421 | 0.99 (0.89-1.10) | 0.836 |
| 40-59.9 y | 10464/49691 | 270732/1356289 | 1.09 (1.06-1.11) | <0.001 |  | 5228/25594 | 94434/472716 | 1.03 (0.99-1.06) | 0.116 |  | 620/3050 | 9181/45955 | 1.03 (0.94-1.13) | 0.530 |
| ≥ 60 y | 13446/70102 | 267750/1335878 | 0.98 (0.96-1.00) | 0.023 |  | 4079/20597 | 95583/477713 | 0.99 (0.95-1.02) | 0.525 |  | 285/1409 | 9516/47596 | 1.01 (0.88-1.16) | 0.880 |
| Age at infection (frequency) |  |  |  |  |  |  |  |  |  |  |  |  |  |  |
| <40 y |  |  |  |  |  |  |  |  |  |  |  |  |  |  |
| 0 | - | 271185/1373845 | Ref. |  |  | - | 97097/486673 | Ref. |  |  | - | 9289/46421 | Ref. |  |
| 1 | 7393/25675 | - | 1.56 (1.52-1.61) | <0.001 |  | 1951/9113 | - | 1.09 (1.03-1.15) | 0.001 |  | 386/1934 | - | 0.99 (0.88-1.11) | 0.874 |
| ≥ 2 | 2618/6460 | - | 2.22 (2.12-2.33) | <0.001 |  | 614/2524 | - | 1.26 (1.15-1.38) | <0.001 |  | 126/650 | - | 0.98 (0.81-1.20) | 0.873 |
| 40-59.9 y |  |  |  |  |  |  |  |  |  |  |  |  |  |  |
| 0 | - | 270732/1356289 | Ref. |  |  | - | 94434/472716 | Ref. |  |  | - | 9181/45955 | Ref. |  |
| 1 | 8220/38861 | - | 1.09 (1.06-1.12) | <0.001 |  | 3983/19780 | - | 1.01 (0.98-1.05) | 0.559 |  | 470/2329 | - | 1.02 (0.92-1.14) | 0.655 |
| ≥ 2 | 2244/10830 | - | 1.07 (1.03-1.13) | 0.002 |  | 1245/5814 | - | 1.08 (1.01-1.15) | 0.020 |  | 150/721 | - | 1.05 (0.88-1.26) | 0.602 |
| ≥ 60 y |  |  |  |  |  |  |  |  |  |  |  |  |  |  |
| 0 | - | 267750/1335878 | Ref. |  |  | - | 95583/477713 | Ref. |  |  | - | 9516/47596 | Ref. |  |
| 1 | 10145/52408 | - | 0.98 (0.96-1.01) | 0.165 |  | 3096/15532 | - | 0.99 (0.96-1.04) | 0.798 |  | 222/1073 | - | 1.04 (0.89-1.20) | 0.649 |
| ≥ 2 | 3301/17694 | - | 0.96 (0.92-0.99) | 0.022 |  | 983/5065 | - | 0.97 (0.90-1.04) | 0.381 |  | 63/336 | - | 0.93 (0.71-1.22) | 0.597 |
| AD: Alzheimer’s disease; ALS: amyotrophic lateral sclerosis; CI, confidence interval; OR, odds ratio; PD: Parkinson’s disease. Conditional on matching factors (sex and year of birth) and further adjusted for area of residence, educational attainment, family history of the disease, and history of comorbidity. Infections diagnosed during ten years before the index date were excluded to alleviate the potential influence of reverse causation due to diagnostic delay. | | | | | | | | | | | | | | |
