# Supplementary material for: Hospital-treated infections in early- and mid-life and risk of Alzheimer’s disease, Parkinson’s disease, and amyotrophic lateral sclerosis: A nationwide nested case-control study in Sweden
Source: PLoS Med. 2022 Sep 15;19(9):e1004092. doi: 10.1371/journal.pmed.1004092 (PMC9477309; doi:10.1371/journal.pmed.1004092)
Supplement: S1 Text — (DOCX) [file pmed.1004092.s011.docx]

**Statistical Analysis Plan**

**Hospital-treated infections in early- and mid-life and risk of Alzheimer’s disease, Parkinson’s disease, and amyotrophic lateral sclerosis: A nationwide nested case-control study in Sweden**

Jiangwei Sun

IMM, KI

Stockholm, Sweden

**Modification history**

Created by Jiangwei Sun: 2021-09-06

Updated by Jiangwei Sun: 2021-11-23

Updated by Jiangwei Sun: 2022-05-16 (adding non-prespecified analyses according to comments from external reviewers)

**STUDY OBJECTIVES**

The primary objective is to investigate the associations between any hospital-treated infections and risk of AD, PD, and ALS.

The secondary objective is to explore the associations by infection characteristics, including infection type, infection site, timing of infection, and infection frequency.

**METHOD**

**Study design**

We will follow all individuals born after 1900 in Sweden whose parents were also born in Sweden from 1970 until neurodegenerative disease diagnosis, death, first migration out of Sweden, or December 31, 2016, through cross-linkages several Swedish national healthcare registers using the unique personal identity number. The registers include the National Patient Register (NPR), the Total Population Register, the Migration Register, the Causes of Death Register, the Swedish Multi-Generation Register, and the Swedish Longitudinal Integrated Database for Health Insurance and Labor.

Individuals with newly-diagnosed neurodegenerative disease during the follow up will be identified from NPR using the ICD codes in Table 1. Date of diagnosis will be defined as the date of first hospital visit concerning the disease. Individuals with multiple neurodegenerative diseases will contribute to the analyses of different diseases.

Based on the above study base, we will then conduct a nested case-control study to explore associations between hospital-treated infections and risk of neurodegenerative disease. Five controls that are individually matched to each case by sex and year of birth will be randomly selected from the study base by using incidence density sampling. Date of diagnosis for the case and date of selection for the control will be used as the index date. Controls should be alive and free of neurodegenerative disease when being selected.

**Exposure definition and measures**

Hospital-treated infections before the index date will be identified from NPR using the ICD codes in Table 2. It will be categorized by: (i) yes/no; (ii) infection types (e.g., bacterial, viral, and others); (iii) infection sites (e.g., CNS, gastrointestinal, genitourinary, respiratory, and skin); (iv) age at infections (<40, 40-59.9, or ≥60 years), and (v) infection frequency (0, 1, ≥2 events).

**Covariates**

In addition to age and sex, the following potential confounders will be considered. We will link the nested case-control study to the Swedish Longitudinal Integrated Database for Health Insurance and Labor to obtain area of residence (3 groups: Northern, Central, and Southern Sweden) and educational attainment (4 groups: 0-9 years, 10-12 years, ≥13 years, and “missing”). We will calculate the Charlson comorbidity index (a proxy for general health status) before the index date through NPR. Family history of neurodegenerative disease (yes/no) will be defined as a diagnosis of the disease among first-degree relatives (i.e., biological parents and full siblings), and parents and full siblings will be identified from the Swedish Multi-Generation Register.

**STATISTICAL ANALYSES**

We will perform separate analyses for AD, PD, and ALS. We will first describe the percentage of individuals with a history of infections among cases and controls during 20 years before index date and then apply conditional logistic regression to estimate odds ratio (OR) and 95% confidence interval (CI), as an estimate of the association.

In addition to conditioning on matching variables (sex and year of birth), we will adjust for area of residence, educational attainment, family history of neurodegenerative disease, and comorbidity in the analysis. Due to the concern about diagnostic delay of neurodegenerative diseases. We will use a lag time of 5 years, namely infections experienced during 5 years before the index date will be excluded from the main analysis.

The analyses will be first performed for any infection and then by type, site, and age at infection (<40 y, 40-59.9 y, and ≥60 y). Further, to examine a potential dose-response relationship within specific age windows, we will analyze frequency of infections (0, 1, and ≥2 events) by age at infection. For any and specific infection, we will stratify the analysis by sex (male or female) and age at index date (<60 y or ≥60 y) to assess whether associations would differ between male and female or young and older individuals. Because NPR reaches nationwide coverage for inpatient care from 1987 onward, for any infection, we will also stratify the analysis by calendar period (1970-1986 vs 1987-2016) to estimate the impact of register coverage on results. To assess whether associations would be modified by family history of neurodegenerative disease, we will restrict the analysis to those without a family history of the disease. Further, to minimize potential misclassification of outcomes, we will restrict the analysis to those with at least two hospital visits concerning the same disease. Finally, to assess robustness of results, we will perform another sensitivity analysis by excluding infections experienced during 10 years before index date.

Data analyses were performed using SAS version 9.4 (SAS Institute Inc, Cary, NC) and R version 3.6.0. A two-sided P ≤ 0.05 was considered statistically significant.

*Non-prespecified Analysis (Analysis according to comments from external reviewers):*

- To explore whether the associates differ between different calendar periods, we further stratify the calendar period into three groups (i.e., 1970-1986, 1987-2000, and 2001-2016).
- To assess whether associations would differ between different birth cohort, we add a subgroup analysis by year of birth (1900-1919, 1920-1939, 1940-1959, and ≥1960).
- To assess the robustness of the main result, one sensitivity analysis is added by excluding those with multiple neurodegenerative diseases from analysis.
- We add a sensitivity analysis to explore the association between hospital-treated infection and risk of early onset AD and late onset AD.
- Because the positive associations were mainly noted for those diagnosed before 60 years, and the etiologies for AD and PD diagnosed at relatively young age are potentially different from those diagnosed at later age, we repeated all analyses stratified by age at index date.
- To assess whether missing value in education would affect the associations, we repeat the main analyses by not adjusting for education and additionally compared the results between two models with or without adjustment for education among individuals with data available on education.

| Table 1. The Swedish revisions of International Classification of Diseases (ICD) codes for neurodegenerative diseases | | | |
| --- | --- | --- | --- |
| Disease | ICD-8 (1969-1986) | ICD-9 (1987-1996) | ICD-10 (1997-) |
| Alzheimer's disease | 290 | 290A, 290B, 331A | F00, G30 |
| Parkinson's disease | 342 | 332A | G20 |
| Amyotrophic lateral sclerosis | 348,00 | 335C | G122 |

| Table 2: The Swedish revisions of International Classification of Diseases (ICD) codes for hospital-treated infections | | | |
| --- | --- | --- | --- |
|  | ICD-8 | ICD-9 | ICD-10 |
| **Infection type** | | | |
| Bacterial | 000, 001, 002, 003, 004, 005, 073, 076, 080, 081, 082, 083, 320, 362, 380, 381, 382, 383, 421, 461, 481, 482, 501, 510, 567, 590, 595, 597, 612, 613, 614, 616, 620, 622, 630, 635, 670, 678, 680, 681, 682, 684, 710, 720, 010, 011, 012, 013, 014, 015, 016, 017, 018, 019, 020, 021, 022, 023, 024, 025, 026, 027, 030, 031, 032, 033, 034, 035, 036, 037, 038, 039, 090, 091, 092, 093, 094, 095, 096, 097, 098, 099, 100, 101, 102, 103, 104, 390, 391, 392 | 001, 002, 003, 004, 005, 073, 076, 077, 078, 079, 080, 081, 082, 083, 320, 381, 382, 383, 383, 421, 461, 475, 481, 482, 510, 567, 590, 595, 597, 670, 730, 010, 011, 012, 013, 014, 015, 016, 017, 018, 020, 021, 022, 023, 024, 025, 026, 027, 030, 031, 032, 033, 034, 035, 036, 037, 038, 039, 040, 041, 090, 091, 092, 093, 094, 095, 096, 097, 098, 099, 100, 101, 102, 103, 104, 390, 391, 392, 614, 615, 616, 680, 681, 682, 683, 684, 685, 686 | A00, A01, A02, A03, A04, A05, A15, A16, A17, A18, A19, A20, A21, A22, A23, A24, A25, A26, A27, A28, A30, A31, A32, A33, A34, A35, A36, A37, A38, A39, A40, A41, A42, A43, A44, A45, A46, A47, A48, A49, A50, A51, A52, A53, A54, A55, A56, A57, A58, A65, A66, A67, A68, A69, A70, A71, A72, A73, A74, A75, A76, A77, A78, A79, B95, B96, G00, G01, H60, H70, I00, I01, I02, I33, J01, J13, J14, J15, J36, J86, K65, L00, L01, L02, L03, L04, L05, L06, L07, L08, M00, M86, N30, N34, N70, N71, N72, N73, N74, N75, N76, N77, O23, O85, O86, P36 |
| Viral | 075, 360, 420, 422, 460, 464, 465, 466, 480, 040, 041, 042, 043, 044, 045, 046, 050, 051, 052, 053, 054, 055, 056, 057, 060, 061, 062, 063, 064, 065, 066, 067, 068, 070, 071, 072, 073, 074, 075, 076, 077, 078, 079, 470, 471, 472, 473, 474 | 070, 071, 072, 074, 075, 077, 078, 079, 372, 420, 422, 460, 464, 465, 466, 480, 487, 647, 711, 045, 046, 047, 048, 049, 050, 051, 052, 053, 054, 055, 056, 057, 060, 061, 062, 063, 064, 065, 066 | A08, A60, A80, A81, A82, A83, A84, A85, A86, A87, A88, A89, A90, A91, A92, A93, A94, A95, A96, A97, A98, A99, B00, B01, B02, B03, B04, B05, B06, B07, B08, B09, B15, B16, B17, B18, B19, B20, B21, B22, B23, B24, B25, B26, B27, B28, B29, B30, B31, B32, B33, B34, B97, B99, H10, I30, I40, J00, J04, J05, J06, J10, J12, J20, J21, O98, P35, Z21 |
| Others | 084, 085, 086, 087, 088, 089, 112, 113, 114, 117, 120, 121, 122, 124, 125, 126, 128, 130, 131, 132, 133, 134, 135, 136, 363, 610, 611, 615, 732, 763, Y41 | 084, 085, 086, 087, 088, 112, 113, 114, 117, 118, 120, 121, 122, 124, 125, 126, 128, 130, 131, 132, 133, 134, 135, 136, 370, 675, 771, 137, 138, 139 | V02, B37, B38, B45, B46, B47, B48, B49, B50, B51, B52, B53, B54, B55, B56, B57, B58, B60, B61, B62, B63, B64, B65, B66, B67, B68, B69, B70, B72, B73, B74, B75, B76, B77, B78, B79, B80, B83, B85, B86, B87 B88, B89, B90, B91, B92, B93, B94, H16, H32, M01, M02, M03, O91, P37, P38, P39, Z22, A59, A63, A64 |
| **Infection site** | | | |
| CNS | 013, 062, 063, 064, 065, 066, 071, 094, 292, 320, 323, 324, 390, 474, 040, 041, 042, 043, 044, 045, 046 | 013, 062, 063, 064, 071, 094, 320, 323, 326, 392, 045, 046, 047, 048, 049 | A17, A80, A81, A82, A83, A84, A85, A86, A87, A88, A89, G00, G01, G02, G04, G05, I02 |
| Gastrointestinal | 014, 123, 127, 129, 540, 567, 000, 001, 002, 003, 004, 005, 006, 007, 008, 009 | 014, 123, 127, 129, 540, 567, 000, 001, 002, 003, 004, 005, 006, 007, 008, 009 | A00, A01, A02, A03, A04, A05, A06, A07, A08, A09, B71, B81, B82, K35, K65, K67 |
| Genitourinary | 016, 590, 595, 597 | 016, 590, 595, 597 | N30, N34, O23, N390 |
| Respiratory | 010, 011, 012, 033, 034, 075, 115, 116, 490, 501, 503, 510, 460, 461, 462, 463, 464, 465, 466, 470, 471, 472, 473, 474, 480, 481, 482, 483, 484, 485, 486 | 010, 011, 012, 033, 034, 075, 115, 116, 473, 475, 487, 490, 510, 460, 461, 462, 463, 464, 465, 466, 480, 481, 482, 483, 484, 485, 486 | A15, A16, A37, A38, B27, B39, B40, B41, B42, B44, B59, J00, J01, J02, J03, J04, J05, J06, J10, J12, J13, J14, J15, J16, J17, J18, J20, J21, J22, J32, J36, J40, J41, J42, J86, P23 |
| Skin | 110, 111, 050, 051, 052, 053, 054, 055, 056, 057, 680, 681, 682, 683, 684, 685, 686 | 110, 111, 050, 051, 052, 053, 054, 055, 056, 057, 680, 681, 682, 683, 684, 685, 686 | B00, B01, B02, B03, B04, B05, B06, B07, B08, B09, B35, B36, B43, L00, L01, L02, L03, L04, L05, L06, L07, L08 |
