# Supplementary material for: Hospital-treated infections in early- and mid-life and risk of Alzheimer’s disease, Parkinson’s disease, and amyotrophic lateral sclerosis: A nationwide nested case-control study in Sweden
Source: PLoS Med. 2022 Sep 15;19(9):e1004092. doi: 10.1371/journal.pmed.1004092 (PMC9477309; doi:10.1371/journal.pmed.1004092)
Supplement: Attachment — Submitted filename: letter to editor and reviewer.docx [file pmed.1004092.s012.docx]

**Manuscript ID number:** PMEDICINE-D-22-01054R1

**Title of paper (after revision):** Hospital-treated infections in early- and mid-life and risk of Alzheimer’s disease, Parkinson’s disease, and amyotrophic lateral sclerosis: a nationwide nested case-control study in Sweden

**Requests from the editors:**

Please revisit the data statement (submission form) to detail the source(s) of data and provide contact information for readers interested in seeking access.

**Reply:** Thank you for this suggestion. We have now revised the data statement in the submission form as follows:

*The data set cannot be shared directly under current legislation for data protection and must be requested directly from the respective registry holders, Statistics Sweden (information@scb.se) and the Swedish National Board of Health and Welfare (registerservice@socialstyrelsen.se), after approval by the Swedish Ethical Review Authority.*

Please adapt the title to better match journal style: we suggest "Hospital-treated infections in early- and mid-life and risk of Alzheimer’s disease, Parkinson’s disease and amyotrophic lateral sclerosis: A case-control study".

**Reply:** Thank you for your suggestion. Also considering a comment of reviewer 2, the title has been changed to: “Hospital-treated infections in early- and mid-life and risk of Alzheimer’s disease, Parkinson’s disease, and amyotrophic lateral sclerosis: a nationwide nested case-control study in Sweden”.

Please specify the country in the title.

**Reply:** See above.

Please restructure the abstract to combine the "Methods" and "Findings" subsections.

**Reply:** The two subsections have now been combined.

Please add a new final sentence to the combined subsection, which should begin "Study limitations include ..." or similar and should quote 2-3 of the study's main limitations.

**Reply:** Thank you for the suggestion. We have now stated the main limitations of the study in the combined subsection as follows:

*On page 3 (page number in the version with track changes):*

*“Study limitations include the potential misclassification of hospital-treated infections and neurodegenerative diseases due to incomplete coverage of the National Patient Register, as well as the residual confounding from unmeasured risk or protective factors for neurodegenerative diseases.”*

In the abstract and throughout the paper, please quote p values alongside 95% CI, where available.

**Reply:** The *P* values have now been added as requested.

After the abstract, please include and new and accessible "author summary" section in non-identical prose. You may find it helpful to consult one or two recent research articles published in PLOS Medicine to get a sense of the preferred style.

**Reply:** We appreciate your suggestion. The “author summary” section has now been added after the abstract. Please also see below:

*On pages 4-5:*

***Author summary:***

***Why was this study done?***

*Experimental studies suggest that infection plays a role in neurodegenerative disease development. Supporting evidence in humans is however scarce. Due to the long preclinical stage of neurodegenerative diseases and the short follow-up in existing studies, it is still unclear whether infection constitutes a risk factor or is merely a comorbidity or secondary event. Further, no study has explored the association of infections treated in specialized care (i.e., hospital in- and out-patient care) with the subsequent risk of common neurodegenerative diseases (AD, PD, and ALS) in a single population, with further analysis on infection characteristics (i.e., type, site, age, and frequency of infection).*

***What did the researchers find?***

*After excluding infections experienced within 5 years before diagnosis of AD, PD, or ALS, infections treated in specialized care were associated with an increased subsequent risk of AD (OR=1.16, 95%CI: 1.15-1.18, P < 0.0001) and PD (OR=1.04, 95%CI: 1.02-1.06, P = 0.0001), but not ALS (OR=0.97, 95%CI: 0.92-1.03, P = 0.3838). Increased risks of AD and PD were observed for bacterial, viral, and other infections, and across different sites of infection, including central nervous system, gastrointestinal, and genitourinary infections. Individuals with repeated infections in early- and mid-life had the greatest risk increment of AD and PD. These positive associations remained after excluding infections experienced within 10 years before diagnosis of AD or PD.*

***What do these findings mean?***

*Infections requiring treatment in specialized care in early- and mid-life were associated with an increased risk of AD and PD. The underlying mechanisms are likely not specific to certain pathogens or affected organs but occur at the systemic level. The findings that infections were more strongly associated with the risk of AD and PD before age 60 and that individuals with repeated infections in early- and mid-life had the greatest risk increment suggest that infectious events may be a trigger or amplifier of a pre-existing disease process, leading to clinical onset of neurodegenerative disease at a relatively early age among individuals with disease predisposition. However, due to the observational nature of the study, these results do not formally prove a causal link.*

Early in the Methods section (main text), please state whether or not the study had a protocol or prespecified analysis plan, and if so attach the relevant document as a supplementary file. Please highlight analyses that were not prespecified.

**Reply:** Thank you for the suggestion. We have now stated about the pre-specified analysis plan in Method, added it as a supplementary file (S1 Text), and highlighted analyses that were not pre-specified.

*On page 8:*

*“The pre-specified analysis plan is presented in S1 Text.”*

Throughout the text, please format reference call-outs to the following style: "... genetic causes [6,7]." (noting the absence of spaces within the square brackets). In the reference list, please convert all italics and boldface text into plain text. Where appropriate, please list 6 author names, followed by "et al.". Noting reference 5 and others, please ensure that all references have full access details.

**Reply:** Thank you for your suggestion. The references have been formatted accordingly.

Please include a completed checklist for the most appropriate reporting guideline, e.g., STROBE, as an attachment, labelled "S1_STROBE_Checklist" or similar and referred to as such in the Methods section (main text). In the checklist, please refer to individual items by section, e.g., "Methods" and paragraph number, not by line or page numbers as these generally change in the event of publication.

**Reply:** We appreciate your suggestion and have now revised the STROBE checklist accordingly, referred to it in Method, and attached it as a supplementary file (S2 STROBE Checklist).

*On page 12:*

*“This study is reported according to the Strengthening the Reporting of Observational Studies in Epidemiology (STROBE) guideline (S2 STROBE Checklist).”*

**Comments from the reviewers:**

***** Reviewer #1 (statistical reviewer):**

This study aims to examine the risk of three most common neurodegenerative diseases in relation to previous inpatient or outpatient episodes of hospital-treated infections.

Comments:

"Hospital-treated infections, especially in early and mid-life, were associated with an increased subsequent risk of AD and PD, especially of AD and among cases diagnosed at relatively young age. These findings suggest that infectious events may be a trigger or amplifier of a pre-existing disease process, leading to clinical onset of neurodegenerative disease at a relatively early age."

Can the authors please revisit the conclusions, being mindful that the study and analytical design does not allow causality to be inferred?

**Reply:** Thank you for highlighting this important point. We have now revised the conclusions in Abstract as follows:

*On page 3:*

*“****Conclusions*** *Hospital-treated infections, especially in early- and mid-life, were associated with an increased risk of AD and PD, especially of AD and among cases diagnosed at relatively young age. These findings suggest that infectious events may be a trigger or amplifier of a pre-existing disease process, leading to clinical onset of neurodegenerative disease at a relatively early age. However, due to the observational nature of the study, these results do not formally prove a causal link.”*

"All individuals born after 1900 in Sweden whose parents were also born in Sweden were eligible for this study (N=12,275,551). We followed them from 1970 until a diagnosis of neurodegenerative disease, emigration, death, or December 31st, 2016, whichever occurred first, through cross linkages to the National Patient Register (NPR) and the Causes of Death Register, using the unique personal identity number."

Can the authors please clarify if they did or did not capture cases of onset of neurodegenerative disease before 1970?

**Reply:** Thank you for the comment. We only included newly diagnosed cases after 1970 in the analyses. Individuals with a diagnosis of the three neurodegenerative diseases before 1970 were excluded from the analysis. We have now clarified this in Method.

*On page 8:*

*“Individuals with a diagnosis of neurodegenerative diseases before 1970 were excluded from the analyses, and individuals with multiple neurodegenerative diseases during follow-up contributed to the analyses of different diseases.”*

Can the authors please comment on whether there may have been a change in diagnosis process and/or accuracy over time that could be impacting on the study inferences? Similarly, whether changes in individuals' capacity to treat infections over time might have an affect on the study findings?

**Reply:** We appreciate the reviewer’s comment and has now added text in Discussion to address this.

*On page 22:*

*“In the present study, although positive associations were observed for AD and PD diagnosed before age 60 and for infections in early- and mid-life throughout the study period, the magnitude of the associations decreased over time. There are likely multiple explanations. First, the treatment of infections might have changed during the study period, with presumably better treatment outcomes during later calendar periods than before. Similarly, the diagnosis of neurodegenerative diseases might have improved over time, not only thanks to improvements in diagnostic tools, especially imaging and more recently also soluble biomarkers, but also due to improved societal awareness of these diseases and resources in neurology and geriatric care. Second, we included outpatient care data for the definition of hospital-treated infections since 2001, whereas only inpatient care data were available before then. Infections requiring specialized care, especially inpatient care, might have a different role on neurodegenerative diseases compared to infections not requiring such. Indeed, a previous study suggested a much weaker association between primary care-based infections, compared with hospital-treated infections, and the risk of dementia (hazard ratio=1.02; 95%CI: 1.00-1.04 vs. 1.99; 95%CI:1.94-2.04).[18]”*

"The analysis included 291,941 AD cases (median age at diagnosis: 76.2 years; male: 46.6%), 103,919 PD cases (74.3; 55.1%), and 10,161 ALS cases (69.3; 56.8%)."

Can the authors please comment on whether the analysed samples can be considered to be representative of the wider populations of interest?

**Reply:** Thank you for the comment. The studied population consisted of all patients with a newly diagnosed neurodegenerative disease during 1970-2016 in Sweden. As suggested in previous validation studies [ref 1, ref 2, ref 3], the Swedish National Patient Register is a valid data source for epidemiological studies of neurodegenerative diseases. However, as all cases were identified through specialist care, there is a delay comparing the register-based date of diagnosis (especially for the ones identified through inpatient care) and the actual date of diagnosis. Also, although the studied neurodegenerative diseases should theoretically all be referred to specialized care by a neurologist or geriatrician, we cannot exclude the possibility that some patients might have only been attended by primary care and thus misclassified as free of these diseases in the National Patient Register. We have now discussed this in Discussion.

*On page 25:*

*“The patient population included in the study consisted of all patients with a newly diagnosed neurodegenerative disease during 1970-2016 in Sweden. As suggested in previous validation studies,[23-25] the National Patient Register is a valid data source for epidemiological studies of neurodegenerative diseases. However, as all cases were identified through specialist care, there was likely a delay comparing the register-based date of diagnosis (especially for the ones identified through inpatient care) and the actual date of diagnosis. Also, although the studied neurodegenerative diseases should theoretically all be referred to specialized care by a neurologist or geriatrician, we cannot exclude the possibility that some patients might have only been attended in primary care and misclassified as not having the disease in the National Patient Register.”*

**Ref 1:** Feldman A L, Johansson A L, Gatz M, et al. Accuracy and sensitivity of Parkinsonian disorder diagnoses in two Swedish national health registers[J]. Neuroepidemiology, 2012, 38(3): 186-93.

**Ref 2:** Rizzuto D, Feldman A L, Karlsson I K, et al. Detection of Dementia Cases in Two Swedish Health Registers: A Validation Study[J]. J Alzheimers Dis, 2018, 61(4): 1301-1310.

**Ref 3:** Mariosa D, Hammar N, Malmstrom H, et al. Blood biomarkers of carbohydrate, lipid, and apolipoprotein metabolisms and risk of amyotrophic lateral sclerosis: A more than 20-year follow-up of the Swedish AMORIS cohort[J]. Ann Neurol, 2017, 81(5): 718-728.

"Individuals with multiple neurodegenerative diseases contributed to the analyses of different diseases."

Did the authors consider conducting a sensitivity analysis excluding individuals with multiple neurodegenerative diseases within each analysis?

**Reply:** Thank you for your suggestion. We have now performed a sensitivity analysis with exclusion of individuals with multiple diseases, where we observed very similar results. This has now been added to Method and Result, respectively.

*Method, on page 11:*

*“5) We additionally excluded individuals with more than one neurodegenerative diseases from the analysis (2.64% with AD and PD, 0.05% with AD and ALS, 0.04% with PD and ALS, and <0.01% with all three diseases).”*

*Result, on page 19:*

*“5) Similar results were also observed after excluding individuals with multiple neurodegenerative diseases, i.e., the OR of AD, PD and ALS in relation to any hospital-treated infection was 1.17 (95%CI: 1.16-1.19, P < 0.0001), 1.04 (95%CI: 1.02-1.06, P = 0.0003), and 0.97 (95%CI: 0.92-1.03, P = 0.3732), respectively.”*

"Five controls per case, individually matched by sex and year of birth, were randomly selected from the study base using incidence density sampling method." A rigorous matching methodology has been applied by the authors.

**Reply:** We appreciate the reviewer’s positive feedback.

"We then applied conditional logistic regression to estimate odds ratio (OR) and 95% confidence 135 interval (CI), as an estimate of the association."

The authors have applied technically appropriate modelling methods within the context of this research.

**Reply:** We appreciate the reviewer’s positive comment.

"Due to the concern about diagnostic delay, we used a lag time of 5 years, namely infections experienced during 5 years before the index date were excluded, in the main analysis. "

Can the authors please clarify how individuals with multiple cases of infection (<= and > 5 years before diagnosis) were accounted for in the study? i.e. was the infection excluded if within five years of diagnosis, or the individual?

**Reply:** Thank you for this comment. Only events of hospital-treated infections, rather than individuals, during 5 years before the index date were excluded. For example, an individual with two infection events occurring at 8 and 3 years before the index date would not be excluded from the analysis, although only the event that occurred at 8 years before the index date will be taken into consideration in the analysis. We have now clarified this in Method.

*On page 10:*

*“Due to the concern of surveillance bias and reverse causation,[23,24,33] we used a lag time of 5 years, namely all infections experienced during 5 years before the index date, were excluded from the analysis.”*

The authors have undertaken a thorough and extensive array of secondary and sensitivity analyses, which help to demonstrate the robustness of the study findings.

**Reply:** Thank you for your positive comment.

The Results are presented accurately and the main study limitations are transparently discussed.

**Reply:** We are grateful for your encouragement.

***** Reviewer #2:**

This is a case-control study nested within national Swedish registers examining the associations of hospital-treated infections with Alzheimer's disease, Parkinson's disease and amyotrophic lateral sclerosis. The study is interesting and timely and benefits from a very large dataset. I also commend the authors for 5- and 10-year lag analyses to reduce the risk of reverse causation and ascertainment bias. My suggestions to improve the study are as follows:

**Reply:** We appreciate your positive assessment.

Major points:

Because this is an observational study, I would suggest using more cautious language in the title. For example, "associated with an increased risk" would be more appropriate than "increase the risk", which implies causality. The title would perhaps also be more informative if it mentioned ALS as well.

**Reply:** Thank you for your comment. After taking into account both this comment and a comment of the editors, we have now changed the title as “Hospital-treated infections in early- and mid-life and risk of Alzheimer’s disease, Parkinson’s disease, and amyotrophic lateral sclerosis: a nationwide nested case-control study in Sweden”.

The methods mention the specificity of the register-based outcome event definitions. It would be fair to report their sensitivity as well.

**Reply:** Thank you for the suggestion. After taking into account both this comment and a comment of reviewer 3, we have now added the sensitivity and positive predictive value in Method.

*On page 8:*

*“The register-based definitions of AD, PD, and ALS have been validated against gold-standard clinical workup, showing a high specificity but a varying sensitivity and positive predictive value for AD (99.7%, 32.5%, and 57%),[23] PD (>98%, 72%, and 71%),[24] and ALS (all >90%).[25]”*

Regarding Table 1: I would assume the characteristics were recorded at index date rather than baseline. Please clarify.

**Reply:** Thank you for bringing this to our attention. The characteristics were indeed recorded at the index date. We have now clarified this and changed the title of Table 1 into “Table 1. Characteristics of patients with neurodegenerative diseases and their matched controls at the index date”, on page 13.

Please clarify when were each of the covariates recorded. I would say that appropriate times for recording them would be on index date or earlier but not later (to prevent the covariates from being affected by the outcome).

**Reply:** This is now clarified in Method that all covariates were measured on the index date or earlier.

*On page 9:*

*“All covariates were measured on the index date or earlier.”*

Missing information in education was included as a separate category for adjustments. This approach would be fine if missing data were rare, but in this case up to half of the participants had missing information on education, which may lead to confounded estimates.

**Reply:** Thank you for the comment. Because the education data are available only since 1990 in the Swedish Longitudinal Integrated Database for Health Insurance and Labor Market Studies [ref 1], we have a large degree of missing data on education. To address this issue, we categorized individuals with missing information on education into a separate category (i.e., “missing”) when adjusting this covariate in the model. We have now added this information in Method.

*On page 9:*

*“We identified information on area of residence (3 groups: Northern, Central, and Southern Sweden) and educational attainment (4 groups: 0-9 years, 10-12 years, ≥13 years, and “missing”) from the Swedish Longitudinal Integrated Database for Health Insurance and Labor Market Studies.[28]”*

However, we agree with the reviewer’s concern and have now added a sensitivity analysis without adjustment for education to assess the influence of the missingness on the results. Compared to the model that adjusted for education, we observed similar results for AD, PD, and ALS in this new analysis. We have now added this new analysis in Method and Result.

*On page 11:*

*“4) Because information on education was available only from 1990 onward, there was a high degree of missingness for this covariate. To assess to what extent this could influence the results, we repeated the main analysis without adjustment for education.”*

*On page 19:*

*“4) We observed similar results for AD (OR=1.16, 95%CI: 1.15-1.18, P < 0.0001), PD (OR=1.04, 95%CI: 1.02-1.06, P = 0.0001), and ALS (OR=0.97, 95%CI: 0.92-1.04, P = 0.3791) in the model without adjusting for education.”*

**Ref 1:** Ludvigsson J F, Svedberg P, Olen O, et al. The longitudinal integrated database for health insurance and labour market studies (LISA) and its use in medical research[J]. Eur J Epidemiol, 2019, 34(4): 423-437

Please clarify, how was the index date defined in the analysis of at least two outcome events. Was it that of the first or second event? Where are these findings reported?

**Reply:** We appreciate the reviewer’s comment. If one individual was diagnosed with more than one of the studied neurodegenerative diseases, for example a diagnosis of PD in 2008 and a later diagnosis of AD in 2012, this individual would contribute to the analysis of PD and AD with the respective diagnosis date used as the respective index date. We have now clarified this in Method.

*On page 10:*

*“If one individual was diagnosed with more than one of the studied neurodegenerative diseases, for example a diagnosis of PD first and a diagnosis of AD later, this individual would contribute to the analysis of PD and AD with the respective diagnosis date.”*

We have also added a new analysis excluding individuals with multiple outcomes from the analysis, taking into consideration this comment and a comment of Reviewer 1.

*Method, on page 11:*

*“5) We additionally excluded individuals with more than one neurodegenerative diseases from the analysis (2.64% with AD and PD, 0.05% with AD and ALS, 0.04% with PD and ALS, and <0.01% with all three diseases).”*

*Result, on page 19:*

*“5) Similar results were also observed after excluding individuals with multiple neurodegenerative diseases, i.e., the OR of AD, PD and ALS in relation to any hospital-treated infection was 1.17 (95%CI: 1.16-1.19, P < 0.0001), 1.04 (95%CI: 1.02-1.06, P = 0.0003), and 0.97 (95%CI: 0.92-1.03, P = 0.3732), respectively.”*

In the Discussion, the authors write that assessing only hospital-treated infections underestimates true associations. I think overestimation is also possible as hospital-treated infections are obviously more severe than those not requiring hospital treatment. This thought is also supported by a previous study, in which the association of primary care-recorded infections with dementia was very weak (Muzambi et al. Lancet Healthy Longev 2021;2(7):e426-35).

**Reply:** We agree with the reviewer. This has now been implemented in Discussion.

*On page 22:*

*“Infections requiring specialized care, especially inpatient care, might have a different role on neurodegenerative diseases compared to infections not requiring such. Indeed, a previous study suggested a much weaker association between primary care-based infections, compared with hospital-treated infections, and the risk of dementia (hazard ratio=1.02; 95%CI: 1.00-1.04 vs. 1.99; 95%CI:1.94-2.04).[18]”*

*On pages 24-25:*

*“Misclassification of hospital-treated infections and neurodegenerative diseases is another concern. Due to incomplete coverage of inpatient care data before 1987 and lack of outpatient care data before 2001 in the National Patient Register, some individuals with hospital-treated infections might have been misclassified as not having infection. Further, our definition of infections did not include those attended by primary care or not attended by health care at all. As a result, the present findings should only be interpreted in the context of relatively severe infections requiring specialist care.”*

The first sentence of the Discussion states that individuals with neurodegenerative diseases were more likely to experience hospital-treated infections. This is confusing, because only 2 of the 3 assessed neurodegenerative conditions were associated with infections.

**Reply:** Thank you for the comment. We have now revised the sentence to make it more precise. Please see below:

*On page 20:*

*“In this nationwide study, we found that hospital-treated infections 5 years ago or earlier were associated with an increased risk of AD and PD, especially of AD and among cases diagnosed before age 60, but not ALS.”*

Vascular, traumatic and alcohol-related brain damage are common aetiologies of early onset dementia (e.g., McMurtray et al. Dement Geriatr Cogn Disord. 2006;21(2):59-64). Thus, in the interpretation of the findings, it should be taken into account that the observed associations of early and middle age infections with dementia could at least in part be explained by confounding due to vascular risk factors, head traumas or alcohol or other substance problems. Could these factors also be taken into account in the analysis of the data?

**Reply:** This is a very relevant remark. We have added a cautionary note, including the suggested reference, relating to potential confounding by such comorbidity in Discussion. As we do not have the complete list of all diagnoses in the present dataset, we can unfortunately not adjust for these in the analysis.

*On page 26:*

*“Residual confounding is also a concern as we did not have complete information on all risk or protective factors for neurodegenerative diseases, including lifestyle factors (smoking and body mass index), medical factors (e.g., human leucocyte antigen type[52] and brain damage due to vascular, traumatic, and substance abuse-related reasons[53]), and genetic factors (e.g., ethnicity or risk genes).[54]”*

Minor points:

Please clarify why were only those whose parents were born in Sweden included in the study.

**Reply:** We conducted this study by using data from a larger project on comorbidity patterns of human diseases using the Swedish national healthcare register data. In this larger project, the register holders in Sweden only gave us access to a population that were born in Sweden and with both parents born in Sweden.

The statistical analysis section states that ORs from nested case-control studies mimic relative risk estimates of the underlying cohort study. To my understanding, this depends on the sampling method used unless the outcome is rare.

**Reply:** Thank you for the comment. To the best of our knowledge, if controls are selected by the method of incidence density sampling, the odds ratio (OR) derived from a nested case-control study is mathematically equal to relative risk estimate in the underlying cohort, and, for this, no rare disease assumption is needed [ref 1]. We have now clarified this in Method.

*On page 10:*

*“As OR obtained from a nested case-control study within a well-defined underlying cohort and with controls selected using the method of incidence density sampling is mathematically equal to relative risk estimate of the underlying cohort study,[31] OR in present study can be interpreted as incidence rate ratio.[32]”*

Ref 1: Greenland, Sander, and Duncan C. Thomas. "On the need for the rare disease assumption in case-control studies." American journal of epidemiology 116.3 (1982): 547-553.

Although most previous studies focused on specific infections, this is not true of all the previous studies cited in the manuscript, although the first sentence of the strengths and limitations section of the Discussion (p. 16) seems to imply so.

**Reply:** We agree with the reviewer and have now revised the first sentence accordingly.

*On page 23:*

*“Unlike most previous studies, our study did not focus on specific infectious diseases (e.g., influenza, pneumonia, or viral hepatitis), but instead studied all infections requiring hospital treatment.”*

Discussion, p. 17, lines 317-319: I agree that the register-based approach does not capture all patients with neurodegenerative diseases. However, contrary to what the text implies, this cannot necessarily be deduced from the low positive predictive values, but is rather dependent on the sensitivity of the outcome ascertainment procedure.

**Reply:** We appreciate the reviewer’s comment. We have revised the sentence accordingly.

*On page 25:*

*“In addition, as the sensitivity of register-based definition is low for AD (32.5%),[23] although higher for PD (72%)[24] and ALS (>90%)[25], not all patients with neurodegenerative diseases were identified, which might have diluted the real associations toward null.”*

The manuscript would benefit from language checking.

**Reply:** Thank you for the suggestion. We have now edited the manuscript thoroughly as requested.

***** Reviewer #3:**

Summary

This was a clearly-written paper that used a nested matched case control design in Swedish National Patient Register data to compare the likelihood of hospitalised infections occurring more than 5 years prior to diagnosis between 291,941 cases of AD, 103,919 of PD or 10,161 of ALS and up to 5 controls per case matched on age and sex. Prior hospital-treated infections were more common among AD cases (1.16, 95% C.I. 1.15-1.18) and PD cases (1.04, 95% C.I. 1.02-1.06) but not ALS cases (OR 0.97, 95% C.I. 0.92-1.03). Associations were stronger for younger patients, and those with multiple infection episodes.

**Reply**: Thank you for the positive comment.

Originality/ importance

Whether infections are a causal risk factor for neurodegenerative diseases is an important question with scope for major health impact. Existing longitudinal studies of clinically symptomatic infections and dementia have tended to focus on limited infection types e.g. sepsis or pneumonia [1,2]. There have been two recent large cohort studies from the UK and Finland, which showed positive associations between broad groups of medically-diagnosed infections and incident dementia risk [3,4]. This study extends findings to a different setting, with longer follow up and investigates two other neurodegenerative conditions, ALS and PD, as well as Alzheimer's disease.

References

1. Muzambi R et al. J Alzheimers Dis. 2020;76(4):1609-1626

2. Chu CS et al. Brain Behav Immun. 2022 Apr 4;103:12-18

3. Muzambi R et al. Lancet Healthy Longev. 2021 Jul;2(7):e426-e435

4. Sipilä PN et al. Lancet Infect Dis. 2021 Nov;21(11):1557-1567

**Reply:** We appreciate your positive assessment.

Specific comments:

1. In the methods section on p6, high figures are given for the specificity of register-based definitions of the three outcomes. It would be helpful to include figures for other aspects of validity such as sensitivity and positive predictive value, especially as the potential for outcome misclassification is later suggested as a possible limitation.

**Reply:** Thank you. We have now added this information in the Method.

*On page 8:*

*“The register-based definitions of AD, PD, and ALS have been validated against gold-standard clinical workup, showing a high specificity but a varying sensitivity and positive predictive value for AD (99.7%, 32.5%, and 57%),[23] PD (>98%, 72%, and 71%),[24] and ALS (all >90%).[25]”*

2. The study time period begins in 1970. While this is a strength in terms of length of follow up, there have presumably been major changes in diagnostic and recording practices for exposures, outcomes and covariates over that time period. Creating time strata of 1970-1986 and 1987-2016 may not be sufficient to highlight differences in results in different time periods. Did you consider stratifying further? Although there was some discussion of data completeness over time, it would be useful to consider this issue further.

**Reply:** We appreciate your comment. We have now performed a new stratified analysis by calendar period (in three groups, i.e., 1970-1986, 1987-2000, and 2001-2016) to explore whether the observed associations differed between different calendar periods. We have described this new analysis in Method, presented the new result as S4 Table and S8 Table (see the below Table 1 adapted from S4 Table and Table 2 adapted from S8 Table), described them in Result, and discussed this finding in Discussion.

*Method, on page 11:*

*“1) To estimate the impact of register coverage and calendar period, we also stratified the analysis by calendar period (1970-1986, 1987-2000, and 2001-2016), and further performed a calendar period specific analysis stratified by age at index date and age at infection separately.”*

*Result, on page 19:*

*“1) A stronger association was noted for any hospital-treated infection during 1970-1986 (OR=1.32, 95%CI: 1.27-1.36 for AD, P < 0.0001; OR=1.14, 95%CI: 1.08-1.20 for PD, P < 0.0001), compared to later (S4 Table). Similar result pattern was also seen when further analyzing calendar period-specific associations by age at index date and age at infection (S8 Table).”*

*Discussion, on page 22:*

*“In the present study, although positive associations were observed for AD and PD diagnosed before age 60 and for infections in early- and mid-life throughout the study period, the magnitude of the associations decreased over time. There are likely multiple explanations. First, the treatment of infections might have changed during the study period, with presumably better treatment outcomes during later calendar periods than before. Similarly, the diagnosis of neurodegenerative diseases might have improved over time, not only thanks to improvements in diagnostic tools, especially imaging and more recently also soluble biomarkers, but also due to improved societal awareness of these diseases and resources in neurology and geriatric care. Second, we included outpatient care data for the definition of hospital-treated infections since 2001, whereas only inpatient care data were available before then. Infections requiring specialized care, especially inpatient care, might have a different role on neurodegenerative diseases compared to infections not requiring such. Indeed, a previous study suggested a much weaker association between primary care-based infections, compared with hospital-treated infections, and the risk of dementia (hazard ratio=1.02; 95%CI: 1.00-1.04 vs. 1.99; 95%CI:1.94-2.04).[18]”*

| Table 1. Association between any hospital-treated infection and risk of neurodegenerative disease, stratified analyses by calendar period at diagnosis of neurodegenerative disease | | | |
| --- | --- | --- | --- |
|  | OR (95% CI) | | |
| Groups | AD | PD | ALS |
| Calendar period at diagnosis |  |  |  |
| 1970-1986 | 1.32 (1.27-1.36) | 1.14 (1.08-1.20) | 0.85 (0.67-1.06) |
| 1987-2000 | 1.27 (1.25-1.29) | 1.06 (1.02-1.09) | 0.93 (0.81-1.06) |
| 2001-2016 | 0.98 (0.97-1.00) | 1.00 (0.98-1.03) | 1.00 (0.94-1.08) |
| CI, confidence interval; OR, odds ratio. Conditional on matching factors (sex and year of birth) and further adjusted for area of residence, educational attainment, family history of the disease, and history of comorbidity. Table 2. Subgroup analysis for the association between any hospital-treated infection and risk of neurodegenerative disease, stratified by calendar period at index date and for specific age at index date and age at infection \| \| \|; Groups \| AD \| PD \| ALS; 1970-1986 \| \| \|; Age at index date \| \| \|; **< 60 y** \| **2.39 (2.26-2.52)** \| **1.42 (1.25-1.62)** \| **1.22 (0.80-1.86)**; ≥ 60 y \| 0.94 (0.90-0.98) \| 1.09 (1.02-1.15) \| 0.74 (0.57-0.97); Age at infection \| \| \|; **<40 y** \| **2.80 (2.64-2.96)** \| **1.59 (1.37-1.85)** \| **1.14 (0.58-2.24)**; **40-59.9 y** \| **1.33 (1.22-1.44)** \| **1.16 (1.04-1.28)** \| **1.07 (0.79-1.45)**; ≥ 60 y \| 0.87 (0.83-0.91) \| 1.06 (0.99-1.14) \| 0.58 (0.40-0.84); 1987-2000 \| \| \|; Age at index date \| \| \|; **< 60 y** \| **1.91 (1.86-1.96)** \| **1.59 (1.45-1.75)** \| **0.80 (0.58-1.09)**; ≥ 60 y \| 1.01 (0.99-1.03) \| 0.99 (0.95-1.03) \| 0.95 (0.82-1.11); Age at infection \| \| \|; **<40 y** \| **1.98 (1.92-2.03)** \| **1.80 (1.63-1.98)** \| **0.98 (0.71-1.35)**; **40-59.9 y** \| **1.24 (1.20-1.29)** \| **1.03 (0.97-1.09)** \| **0.84 (0.69-1.03)**; ≥ 60 y \| 0.97 (0.95-0.99) \| 0.98 (0.94-1.02) \| 0.98 (0.81-1.19); 2001-2016 \| \| \|; Age at index date \| \| \|; **< 60 y** \| **1.35 (1.20-1.52)** \| **1.04 (0.95-1.13)** \| **1.06 (0.91-1.23)**; ≥ 60 y \| 0.98 (0.96-0.99) \| 1.00 (0.97-1.03) \| 0.99 (0.91-1.07); Age at infection \| \| \|; **<40 y** \| **1.12 (1.06-1.18)** \| **1.05 (1.00-1.11)** \| **1.00 (0.89-1.12)**; **40-59.9 y** \| **1.06 (1.03-1.08)** \| **1.02 (0.98-1.05)** \| **1.08 (0.98-1.18)**; ≥ 60 y \| 0.94 (0.92-0.96) \| 0.98 (0.95-1.01) \| 0.91 (0.82-1.01); CI, confidence interval; OR, odds ratio. Conditional on matching factors (sex and year of birth) and further adjusted for area of residence, educational attainment, family history of the disease, and history of comorbidity. \| \| \| | | | |

3. Exposure was hospitalised infections occurring at least five years prior to the index date. It was unclear how infections occurring between 5 and 0 years before index were treated, and whether this might lead to exposure misclassification

**Reply:** Thank you for this comment. Only events of hospital-treated infections, rather than individuals, during 5 years before the index date were excluded. For example, an individual with two infection events occurring at 8 and 3 years before the index date would not be excluded from the analysis, although only the event that occurred at 8 years before the index date will be taken into consideration in the analysis. We have now clarified this in Method.

*On page 10:*

*“Due to the concern of surveillance bias and reverse causation,[23,24,33] we used a lag time of 5 years, namely all infections experienced during 5 years before the index date, were excluded from the analysis.”*

Additionally, as we applied this exclusion criterion to both cases and controls, it is not likely to introduce exposure misclassification, especially not differential misclassification.

4. For the AD analysis, over half of participants had missing data on education. How was this dealt with in the analysis?

**Reply:** Thank you for the comment. Because the education data are available only since 1990 in the Swedish Longitudinal Integrated Database for Health Insurance and Labor Market Studies [ref 1], we have a large degree of missing data on education. To address this issue, we categorized individuals with missing information on education into a separate category (i.e., “missing”) when adjusting this covariate in the model. We have now added this information in Method.

*On page 9:*

*“We identified information on area of residence (3 groups: Northern, Central, and Southern Sweden) and educational attainment (4 groups: 0-9 years, 10-12 years, ≥13 years, and “missing”) from the Swedish Longitudinal Integrated Database for Health Insurance and Labor Market Studies.[28]”*

However, we agree with the reviewer’s concern and have now added a sensitivity analysis without adjustment for education to assess the influence of the missingness on the results. Compared to the model that adjusted for education, we observed similar results for AD, PD, and ALS in this new analysis. We have now added this new analysis in Method and Result.

*On page 11:*

*“4) Because information on education was available only from 1990 onward, there was a high degree of missingness for this covariate. To assess to what extent this could influence the results, we repeated the main analysis without adjustment for education.”*

*On page 19:*

*“4) We observed similar results for AD (OR=1.16, 95%CI: 1.15-1.18, P < 0.0001), PD (OR=1.04, 95%CI: 1.02-1.06, P = 0.0001), and ALS (OR=0.97, 95%CI: 0.92-1.04, P = 0.3791) in the model without adjusting for education.”*

**Ref 1:** Ludvigsson J F, Svedberg P, Olen O, et al. The longitudinal integrated database for health insurance and labour market studies (LISA) and its use in medical research[J]. Eur J Epidemiol, 2019, 34(4): 423-437

5. Models were adjusted for a relatively limited number of confounding factors, which might have led to residual confounding. While a lack of data on factors such as BMI, smoking, alcohol was mentioned as a limitation, other potential confounding factors such as ethnicity and socioeconomic status were also not included (the latter would be incompletely captured by education or area of residence). In addition, the rationale for the approach to clinical comorbidities was unclear: why create a Charlson comorbidity index rather than describe and adjust for individual comorbidities separately?

**Reply:** We agree with the reviewer’s concern about residual confounding from unmeasured confounders and have extended this discussion in Discussion.

*On page 26:*

*“Residual confounding is also a concern as we did not have complete information on all risk or protective factors for neurodegenerative diseases, including lifestyle factors (smoking and body mass index), medical factors (e.g., human leucocyte antigen type[52] and brain damage due to vascular, traumatic, and substance abuse-related reasons[53]), and genetic factors (e.g., ethnicity or risk genes).[54]”*

We used Charlson comorbidity index as a proxy for general health status for the study participants at the index date. Many diseases were considered when calculating this index, including myocardial infarction, cerebrovascular disease, peripheral vascular disease, pulmonary disease, chronic kidney disease, liver disease, etc. We adjusted for the comorbidity index instead of all these individual comorbidities, keeping in mind the reduced statistical power when adding a lot of covariates in the model, especially when the number of outcome (e.g., ALS) is small and also in the subgroup analysis. The lack of power might make it difficult to disentangle a real signal from noise.

6. Health-seeking behaviour is likely to affect recording of both exposure and outcome. Was it possible to describe, or adjust for, frequency of consultations or healthcare contacts in the different exposure groups?

**Reply:** We appreciate the reviewer’s suggestion and agree that health-seeking behavior might theoretically affect recording of both exposure and outcome.

However, if this indeed contributed much to the observed associations between infections and AD/PD, we should have observed a similar – if not stronger - association among older individuals, compared to younger individuals. However, we found very strong associations among individuals below 60, whereas a weak or null association among those at 60 or above. Further, as the associations were stronger during earlier calendar period when infections were only identified through inpatient care, it also argues against health-seeking behavior as an alternative explanation for the results, as infections requiring inpatient care are much less likely due to health-seeking behaviors.

Moreover, diagnostic delay of neurodegenerative diseases might be a concern when directly adjusting for frequency of consultations or healthcare contacts as proxies of health-seeking behavior in the model. For example, as suggested in previous studies, diagnosis of PD can be preceded by a prodromal phase of years, characterized by different non-motor symptoms (e.g., sleep disorders and constipation) [ref 1]. Therefore, the altered health-seeking behavior observed years before a diagnosis of neurodegenerative diseases may be driven by these prodromal symptoms. Adjustment for such in this scenario might be adjusting for a potential mediator rather than a confounder.

We are however certainly willing to reconsider our position if the reviewer and editors would disagree with us.

**Ref 1:** Poewe W, Seppi K, Tanner C M, et al. Parkinson disease[J]. Nat Rev Dis Primers, 2017, 3: 17013. doi:10.1038/nrdp.2017.13

Minor comments:

1.The title should fully reflect the study - currently ALS is not mentioned.

**Reply:** Thank you for the comment. We have now changed the title as “Hospital-treated infections in early- and mid-life and risk of Alzheimer’s disease, Parkinson’s disease, and amyotrophic lateral sclerosis: a nationwide nested case-control study in Sweden”.

2.Table 1 gave the proportion of the study population with any comorbidities, but it would be better to present Charlson comorbidity index scores (if this was the analytical approach taken)

**Reply:** We appreciate your suggestion. We have now presented the Charlson comorbidity index score in Table 1 accordingly (on page 14).

3.The English language should be carefully checked throughout.

**Reply:** Thank you for the comment. We have edited the manuscript thoroughly as suggested.

***** Reviewer #4:**

"Hospital-treated infections in early and mid-life increase the risk of Alzheimer's disease and Parkinson's disease" (PMEDICINE-D-22-01054R1)

The objective of this paper was to examine "the risk of three most common neurodegenerative diseases in relation to previous inpatient or outpatient episodes of hospital-treated infections." Swedish national data were used, with a follow-up from 1970 until 2016. The results show hospital-treated infections to be associated with a higher risk of Alzheimer's disease (AD) and Parkinson's Disease but not amyotrophic lateral sclerosis.

I have a number of concerns, listed below.

1. The statement "Infection is one of the most discussed potential non-genetic risk factors for neurodegenerative disease" (line 69-70) needs further elaboration as infection does not figure on most compilations of risk factors for neurodegenerative diseases, AD in particular.

**Reply:** Thank you for your suggestion. We have now revised this sentence in Introduction.

*On page 6:*

*“A potential infectious etiology has been hypothesized for neurodegenerative diseases, as findings in animal studies have demonstrated that infectious processes might impact pathogenesis, phenotype, and progression of neurodegenerative disease.[1,7-9] Proposed underlying mechanisms include modulating misfolding and aggregation of pathological proteins, neuroinflammation, and infiltration of peripheral immune cells into the central nervous system (CNS).[1,9,10] The extrapolation of such findings to a human context is however not straightforward.”*

2. In their review of the current status of knowledge the authors state that the role of "diagnostic delay" (line 78) has not been addressed in previous research. It isn't clear what this means, do they mean delay in diagnosis of neurodegenerative diseases? If this is the case, I did not find any analyses on diagnostic delay.

**Reply:** Thank you for the comment. By “diagnostic delay”, we indeed mean the delay in ascertainment of neurodegenerative diseases. In some previous studies that assessed associations between infectious diseases and risk of dementia [refs 1-3] and AD [ref 4], the influence of diagnostic delay and the resultant reverse causation due to such diagnostic delay (i.e., infection as a result of present but undiagnosed dementia/AD instead of a risk factor) was not fully addressed due to limited sample size and follow-up. For example, in Sipilä et al. [ref 1], the authors did not consider diagnostic delay of dementia in the main analysis. Although the authors repeated the analysis after excluding dementia cases occurred during the first 10 years after infection, such analysis was not conducted for AD, probably due to the relatively small number of AD cases (n=1730). We have now clarified this in Introduction.

*On pages 6-7:*

*“Although several studies have also assessed associations between infectious diseases and risk of dementia[17-19] and AD[20], influence of potential surveillance bias (greater-than-expected surveillance of disease after infections) and reverse causation (due to for example diagnostic delay of neurodegenerative diseases) on the associations was not always fully addressed. Therefore, whether infection is indeed a risk factor rather a comorbidity or secondary event of neurodegenerative disease remains unknown.”*

In our study, to address this issue, we applied a lag time of 5 years in all the main analyses and a lag-time of 10 years in a sensitivity analysis. For example, in the 5-year lag-time analysis, we excluded all hospital-treated infections experienced during 5 years before the index date from the analysis. We have described these two analyses in Method and compared their results in Result.

*On page 10:*

*“Due to the concern of surveillance bias and reverse causation,[23,24,33] we used a lag time of 5 years, namely all infections experienced during 5 years before the index date, were excluded from the analysis.”*

*Method, on page 11:*

*“6) Finally, to assess the impact of choice of lag time, we performed another sensitivity analysis by excluding infections experienced during 10 years before the index date.”*

*Result, on pages 19-20:*

*“6) Finally, after excluding infections experienced during 10 years before index date, similar associations were still noted between any and specific hospital-treated infections and risk of AD and PD (S9 Table). For example, for any infection, the OR of AD and PD was 1.18 (95%CI: 1.16-1.19, P < 0.0001) and 1.04 (95%CI: 1.01-1.06, P = 0.0024), respectively. A dose-response relationship was still observed for frequency of infections after using a 10-year lag time (P for trend < 0.0001). The OR of AD and PD was 2.22 (95%CI: 2.12-2.33, P < 0.0001) and 1.26 (95%CI: 1.15-1.38, P < 0.0001) in relation to ≥2 events of infection before age 40 (S10 Table).”*

**Ref 1:** Sipilä PN, Heikkilä N, Lindbohm JV, Hakulinen C, Vahtera J, Elovainio M, et al. Hospital-treated infectious diseases and the risk of dementia: a large, multicohort, observational study with a replication cohort. The Lancet Infectious Diseases. 2021;21(11):1557-67.

**Ref 2:** Muzambi R, Bhaskaran K, Smeeth L, Brayne C, Chaturvedi N, Warren-Gash C. Assessment of common infections and incident dementia using UK primary and secondary care data: a historical cohort study. The Lancet Healthy Longevity. 2021;2(7):e426-e35.

**Ref 3:** Mawanda F, Wallace RB, McCoy K, Abrams TE. Systemic and localized extra-central nervous system bacterial infections and the risk of dementia among US veterans: A retrospective cohort study. Alzheimers Dement (Amst). 2016;4:109-17.

**Ref 4:** Douros A, Santella C, Dell'Aniello S, Azoulay L, Renoux C, Suissa S, et al. Infectious Disease Burden and the Risk of Alzheimer's Disease: A Population-Based Study. J Alzheimers Dis. 2021;81(1):329-38.

3. A major concern with this paper is what it adds to the recent paper by Sipila et al. Lancet Infect Dis 2021 (reference 17 in the manuscript). The measures of exposure and outcome, and conclusion are much the same.

**Reply:** We appreciate the reviewer’s comment. We believe that it is a strength of the present study to corroborate recently emerging data, in addition to several important novel aspects. Compared with the study by Sipila et al., the present study has the following benefits:

a) A longer follow-up, enabling us to fully consider the influence of potential reverse causation due to diagnostic delay (and even the preclinical stage) of neurodegenerative disease, using either a 5- year lag-time analysis or a 10-year lag-time analysis. In the study by Sipila et al., the authors did not address the influence of diagnostic delay in the main analysis, which could overestimate the interested associations and make it difficult to assess whether infection is a risk factor, rather a comorbidity or secondary event, of neurodegenerative disease.
b) A large sample size, enabling us to conduct several informative subgroup analyses and sensitivity analyses. For example, we identified a total of 291,941 AD patients, in contrast to the 1,730 AD patients identified by the study by Sipila et al.
c) In addition to AD, the present study also investigated two other common neurodegenerative diseases (i.e., PD and ALS), and is therefore the first study to assess the role of infections on the three most common neurodegenerative diseases in a single study.
d) Finally, the present study performed a subgroup analysis by age at infection to assess potential high-risk time window and found that individuals with multiply infections in early- and mid-life had the greatest risk increment of AD and PD. This finding is new and important, suggesting that infectious events may be a trigger or amplifier of a pre-existing disease process, leading to clinical onset of neurodegenerative disease at a relatively early age among individuals with disease predisposition.

We have now modified our manuscript to reflect these:

*Introduction, on pages 6-7:*

*“Although several studies have also assessed associations between infectious diseases and risk of dementia[17-19] and AD[20], influence of potential surveillance bias (greater-than-expected surveillance of disease after infections) and reverse causation (due to for example diagnostic delay of neurodegenerative diseases) on the associations was not always fully addressed. Therefore, whether infection is indeed a risk factor rather a comorbidity or secondary event of neurodegenerative disease remains unknown.”*

*Discussion, on page 21:*

*“Another study reported a dose-response relationship between number of infectious diseases and dementia risk.[17] This study however did not explore the effect of age at infection and whether a dose-response relationship existed still after considering the influence of potential reverse causation due to the pre-clinical stage and diagnostic delay of neurodegenerative diseases. Two studies used the same datasets from the UK reported an increased risk of dementia[18] or AD[20] among individuals with infectious diseases, without however reporting a clear dose-response relationship.”*

*Discussion, on page 24:*

*“To our knowledge, it is the first to date to assess the associations of hospital-treated infections - by type, site, age, and frequency - with risks of the three most common neurodegenerative diseases in the same population.”*

4. The use of a life course approach is promoted in the introduction but the authors chose to use a case-control design, this is a pity. Time to event analyses would have suited the data better; furthermore, the choice of controls retrospectively is never straightforward. It would be certainly better to retain everyone in the analyses so that people who die over the follow-up can be censored so that mortality does not bias findings.

**Reply:** Thank you for the comment. We agree with the reviewer that cohort study is more intuitively fitted for the life course approach. However, other study designs, including case-control study, nested case-control study, cross-sectional study, quasi-experimental design, and randomized controlled trial, can also be applied to further our understanding of health and disease from a life course perspective [ref 1].

We could have used the entire cohort of >12 million individuals to address the research questions of the study. However, we recognized that it might be computationally demanding to analyze >12 million individuals with time-varying exposure as the exposure status of the study participants will change multiple times during the long follow-up, including number of infections, as well as type, site, and age of infections.

We applied therefore instead a nationwide nested case-control study in present study. The nested case-control study is conducted within a well-defined cohort, with ascertainment of all cases of the underlying cohort and random sampling of controls using the method of incidence density sampling [ref 2]. If the controls are selected by incidence density sampling, then the odds ratio (OR) derived from a nested case-control study is mathematically equal to relative risk estimate in the underlying cohort [ref 3]. As the cases and controls were individually matched by age and sex, and had the same index date, when we compared the cases and controls, we could make sure that they had the same length of observation window for exposure. It became therefore much more straightforward to compare exposure to different infections during the same time window. We have added more information about the study design in Method.

*On page 10:*

*“As OR obtained from a nested case-control study within a well-defined underlying cohort and with controls selected using the method of incidence density sampling is mathematically equal to relative risk estimate of the underlying cohort study,[31] OR in present study can be interpreted as incidence rate ratio.[32]”*

**Ref 1:** Halfon, Neal, et al. Handbook of life course health development. 2018: p 541-560.

**Ref 2:** Rothman, Kenneth J., Sander Greenland, and Timothy L. Lash. Modern epidemiology. Vol. 3. Philadelphia: Wolters Kluwer Health/Lippincott Williams & Wilkins, 2008: p 122-123.

**Ref 3:** Greenland, Sander, and Duncan C. Thomas. On the need for the rare disease assumption in case-control studies. American journal of epidemiology 116.3 (1982): 547-553.

5. There is no mention of period or cohort effects in the results; it is possible that these play a role, particularly for the exposure of interest.

**Reply:** We appreciate your comment. We have now performed a new stratified analysis by calendar period (in three groups, i.e., 1970-1986, 1987-2000, and 2001-2016) to explore whether the observed associations differed between different calendar periods. We have described this new analysis in Method, presented the new result as S4 Table and S8 Table (see the Table 1 and Table 2 in this file for detailed information), described them in Result, and discussed this finding in Discussion.

*Method, on page 11:*

*“1) To estimate the impact of register coverage and calendar period, we also stratified the analysis by calendar period (1970-1986, 1987-2000, and 2001-2016), and further performed a calendar period specific analysis stratified by age at index date and age at infection separately.”*

*Result, on page 19:*

*“1) A stronger association was noted for any hospital-treated infection during 1970-1986 (OR=1.32, 95%CI: 1.27-1.36 for AD, P < 0.0001; OR=1.14, 95%CI: 1.08-1.20 for PD, P < 0.0001), compared to later (S4 Table). Similar result pattern was also seen when further analyzing calendar period-specific associations by age at index date and age at infection (S8 Table).”*

*Discussion, on page 22:*

*“In the present study, although positive associations were observed for AD and PD diagnosed before age 60 and for infections in early- and mid-life throughout the study period, the magnitude of the associations decreased over time. There are likely multiple explanations. First, the treatment of infections might have changed during the study period, with presumably better treatment outcomes during later calendar periods than before. Similarly, the diagnosis of neurodegenerative diseases might have improved over time, not only thanks to improvements in diagnostic tools, especially imaging and more recently also soluble biomarkers, but also due to improved societal awareness of these diseases and resources in neurology and geriatric care. Second, we included outpatient care data for the definition of hospital-treated infections since 2001, whereas only inpatient care data were available before then. Infections requiring specialized care, especially inpatient care, might have a different role on neurodegenerative diseases compared to infections not requiring such. Indeed, a previous study suggested a much weaker association between primary care-based infections, compared with hospital-treated infections, and the risk of dementia (hazard ratio=1.02; 95%CI: 1.00-1.04 vs. 1.99; 95%CI:1.94-2.04).[18]”*

Further, as the reviewer suggested, we also performed an analysis to explore the effect of birth cohort (i.e., birth year in 1900-1919, 1920-1939, 1940-1959, or ≥1960). We have now described this new analysis in Method, added the result in S4 Table (see below Table 3 adapted from S4 Table), and described the result in Result.

*Method, on pages 10-11:*

*“We stratified the analysis by sex (male or female), age at index date (<60 y or ≥60 y), and year of birth (1900-1919, 1920-1939, 1940-1959, or ≥1960) to assess whether the associations would differ by sex, age, and birth cohort.”*

*Result, on page 17:*

*“The associations were primarily limited to individuals diagnosed below 60 (OR=1.93, 95%CI: 1.89-1.98 for AD, P < 0.0001; OR=1.29, 95%CI: 1.22-1.36 for PD, P < 0.0001), and were stronger among individuals born after 1960 (OR=1.59, 95%CI: 1.52-1.65 for AD, P < 0.0001; OR=1.39, 95%CI: 1.24-1.56 for PD, P < 0.0001).”*

| Table 3. Association between any hospital-treated infection and risk of neurodegenerative disease, stratified analysis by year of birth | | | |
| --- | --- | --- | --- |
|  | OR (95% CI) | | |
| Groups | AD | PD | ALS |
| Year of birth |  |  |  |
| 1900-1919 | 0.97 (0.96-0.99) | 1.02 (0.99-1.06) | 0.91 (0.78-1.06) |
| 1920-1939 | 1.05 (1.03-1.06) | 1.00 (0.97-1.03) | 0.91 (0.83-0.99) |
| 1940-1959 | 1.78 (1.73-1.84) | 1.08 (1.04-1.13) | 1.07 (0.97-1.18) |
| ≥1960 | 1.59 (1.52-1.65) | 1.39 (1.24-1.56) | 1.11 (0.88-1.40) |
| CI, confidence interval; OR, odds ratio. Conditional on matching factors (sex and year of birth) and further adjusted for area of residence, educational attainment, family history of the disease, and history of comorbidity. | | | |

6. In the primary analysis (lines 186-189) it is not clear if the results for men and women were different as statistical tests for difference are not provided.

**Reply:** As the reviewer suggested, we have now added an interaction test and the relevant *P* value for the interaction test in Method and Result, respectively.

*Method, on page 11:*

*“The P value for interaction was calculated using the Wald test for the product terms between infection and a specific covariate.”*

*Result, on page 17:*

*“In the analysis of any hospital-treated infection, a stronger association was observed among male (OR=1.20, 95%CI: 1.18-1.22, P < 0.0001) than female (OR=1.13, 95%CI: 1.12-1.15, P < 0.0001) for AD, but among female (OR=1.07, 95%CI: 1.05-1.11, P < 0.0001) than male (OR=1.01, 95%CI: 0.98-1.03, P = 0.5364) for PD (both P _for interaction_ < 0.0001, Fig 3 and S4 Table).”*

7. Figure 3 shows that the association of infection and AD was confined to young-onset AD (under 60 years). These results are intriguing as the majority of AD cases in the population are late onset AD. Early onset AD is primarily driven by genetic factors and it is surprising to have 26% of cases in this study to be early onset AD. Please clarify the age at AD onset in this group; the median age at onset of AD in the total study population was 76.2 years.

**Reply:** Thank you for highlighting this interesting result. The median age in the group with young-onset AD was 41 years.

The high percentage of early onset AD (i.e., under 60 years) may depend both on biological and technical reasons:

a) It is true that early onset AD is considered driven more by genetic factors than late onset AD, still such factors do not explain more than a large fraction of disease risk, leaving room for additional modifiers. It is also a fact that AD diagnosed at older ages can be confounded by other disease process such as vascular, metabolic, toxic (e.g., alcohol) and general aging phenomena. We therefore find the stronger association for AD diagnosed at younger ages to be of interest, as this strengthens an association of hospital-treated infections with AD disease processes specifically rather than by modulation of comorbid conditions.
b) Relatively young study population. In the present study, individuals born after 1956 contributed 56.4% of the early-onset AD (42855/76023=56.4%). The proportion is much lower among those born before 1956 (33168/248489=13.3%).

We have now discussed about these in Discussion.

*On pages 25-26:*

*“In addition, as we had relatively young individuals in the study, there was a high proportion of neurodegenerative diseases ascertained below age 60. For instance, 26% of the AD individuals were ascertained below 60 (median age at diagnosis: 41; 56.4% of them born after 1956), higher than expected. However, the proportion was 13.3% among individuals born before 1956.”*

8. It would be better to stratify results reported in Figure 4 as a function of early- and late-onset AD (after 65 years).

**Reply:** We appreciate the reviewer’s suggestion. We have now added the suggested analysis in Method, described the result in Result, and added this result in S5 Table (also see below Table 4 adapted from S5 Table). In this subgroup analysis, we found the associations to be stronger for early-onset AD than late-onset AD, but the pattern was same as the one shown in Figure 4.

*Method, on page 11:*

*“As previous studies have used age 65 to define early- and late-onset AD, [34] we additionally performed a stratified analysis of AD by age 65 at index date.”*

*Result, on page 17:*

*“Stronger associations were also observed for AD ascertained before 65, compared with AD ascertained after 65 (S5 Table).”*

| Table 4. Associations between age at hospital-treated infection and the consequent risks of early-onset AD and late-onset AD (5-year lag time) | | |
| --- | --- | --- |
|  | OR (95% CI) | |
| Groups | Early onset AD | Late onset AD |
| Age at infection (yes/no) |  |  |
| <40 y | 1.95 (1.91-2.00) | 1.11 (1.05-1.18) |
| 40-59.9 y | 1.47 (1.40-1.54) | 1.07 (1.05-1.10) |
| ≥ 60 y | - | 0.96 (0.94-0.97) |
| Age at infection (frequency) |  |  |
| <40 y |  |  |
| 0 | Ref. | Ref. |
| 1 | 1.74 (1.70-1.79) | 1.10 (1.03-1.17) |
| ≥ 2 | 2.70 (2.59-2.81) | 1.19 (1.03-1.37) |
| 40-59.9 y |  |  |
| 0 | Ref. | Ref. |
| 1 | 1.41 (1.34-1.49) | 1.08 (1.05-1.11) |
| ≥ 2 | 1.63 (1.50-1.77) | 1.05 (1.00-1.10) |
| ≥ 60 y |  |  |
| 0 | - | Ref. |
| 1 | - | 0.98 (0.96-0.99) |
| ≥ 2 | - | 0.92 (0.90-0.94) |
| CI, confidence interval; OR, odds ratio. The cut-off between early onset and late onset AD is 65 years old. Conditional on matching factors (sex and year of birth) and further adjusted for area of residence, educational attainment, family history of the disease, and history of comorbidity. | | |

9. The first sentence of the discussion (line 230-231) would be better supported using time to event analyses and a wash-out period of 20 years.

**Reply:** Thank you for your suggestion. We used this sentence to describe what we observed in Figure 1 of the manuscript, namely that, compared with controls, there was a slightly higher percentage of individuals with hospital-treated infections among patients with AD or PD, but not ALS, during 20 years before diagnosis.

As suggested by this reviewer and Reviewer 2, we have now revised the sentence as follows:

*On page 20:*

*“In this nationwide study, we found that hospital-treated infections 5 years ago or earlier were associated with an increased risk of AD and PD, especially of AD and among cases diagnosed before age 60, but not ALS.”*
