# Supplementary material for: Hospital-treated infections in early- and mid-life and risk of Alzheimer’s disease, Parkinson’s disease, and amyotrophic lateral sclerosis: A nationwide nested case-control study in Sweden
Source: PLoS Med. 2022 Sep 15;19(9):e1004092. doi: 10.1371/journal.pmed.1004092 (PMC9477309; doi:10.1371/journal.pmed.1004092)
Supplement: Attachment — Submitted filename: Plos Med June 2022.docx [file pmed.1004092.s013.docx]

**"Hospital-treated infections in early- and mid-life and risk of Alzheimer’s disease, Parkinson’s disease, and amyotrophic lateral sclerosis: a nationwide nested case-control study in Sweden" (PMEDICINE-D-22-01054R2)**

The revised version of this manuscript has addressed most of my concerns but I remain unconvinced by the approach adopted by the authors. They chose to show unstratified analyses as the main analyses but the results make no sense because of the number of difference as a function of age at exposure, birth-year, age at onset of the neurodegenerative diseases, and sex. The results in the abstract (OR of 1.16 for AD, and 1.04 for PD) are incorrect as they aggregate results over groups with considerable heterogeneity.

My expertise in in AD so I will comment primarily on and early onset AD is a different from late (after age 65) onset AD, the first is primarily genetic and the latter is sporadic. To combine these in the primary results is incorrect. Furthermore, Table S4 (see extract below) shows no association between infection and onset of AD after 60 years, while the association with early onset AD has an OR of 1.93. Combing them yield an OR of 1.16 but is this reasonable?

Given the large sample size, careful analyses of the subgroups could make a contribution to our understanding of the association between infection and neurodegenerative diseases.

| S4 Table. Association between any hospital-treated infection and risk of neurodegenerative disease, stratified analyses by sex, age at diagnosis, calendar period at diagnosis, and year of birth | | | | | | | | | | | |
| --- | --- | --- | --- | --- | --- | --- | --- | --- | --- | --- | --- |
|  | AD | | |  | PD | | |  | ALS | | |
| Group | Infection (case/control) | No infection (case/control) | OR (95% CI) |  | Infection (case/control) | No infection (case/control) | OR (95% CI) |  | Infection (case/control) | No infection (case/control) | OR (95% CI) |
| Sex |  |  |  |  |  |  |  |  |  |  |  |
| Male | 20908/92009 | 115090/587981 | 1.20 (1.18-1.22) |  | 8748/44067 | 48533/242338 | 1.01 (0.98-1.03) |  | 903/4700 | 4870/24165 | 0.95 (0.88-1.04) |
| Female | 28881/133767 | 127062/645948 | 1.13 (1.12-1.15) |  | 8070/37824 | 38568/195366 | 1.07 (1.05-1.11) |  | 890/4484 | 3498/17456 | 1.00 (0.92-1.09) |
| Age at diagnosis |  |  |  |  |  |  |  |  |  |  |  |
| < 60 y | 13196/37418 | 62827/342697 | 1.93 (1.89-1.98) |  | 2110/8600 | 11209/57995 | 1.29 (1.22-1.36) |  | 430/2125 | 1815/9100 | 1.02 (0.90-1.16) |
| ≥ 60 y | 36593/188358 | 179325/891232 | 1.00 (0.98-1.01) |  | 14708/73291 | 75892/379709 | 1.01 (0.99-1.03) |  | 1363/7059 | 6553/32521 | 0.96 (0.90-1.03) |
