# Supplementary material for: Hospital-treated infections in early- and mid-life and risk of Alzheimer’s disease, Parkinson’s disease, and amyotrophic lateral sclerosis: A nationwide nested case-control study in Sweden
Source: PLoS Med. 2022 Sep 15;19(9):e1004092. doi: 10.1371/journal.pmed.1004092 (PMC9477309; doi:10.1371/journal.pmed.1004092)
Supplement: Attachment — Submitted filename: letter to editor and reviewers_R2_final.docx [file pmed.1004092.s014.docx]

**Manuscript ID number:** PMEDICINE-D-22-01054R2

**Title of paper:** Hospital-treated infections in early- and mid-life and risk of Alzheimer’s disease, Parkinson’s disease, and amyotrophic lateral sclerosis: a nationwide nested case-control study in Sweden

**Requests from the editors:**

We suggest addressing referee 4's comments by reporting some additional analyses, perhaps in the supplementary information.

**Reply:** Thank you for the suggestion. The analyses suggested by the reviewer have now been reported in the manuscript. Please see our detailed response below.

Please reformat the 'Author summary' so that the 3 subsections each consists of around 3 bulleted points, each comprising 1-2 short sentences.

**Reply:** We appreciate your suggestion. The "Author summary" section has now been reformatted as requested. Please also see below:

*On pages 4-5 (page number in the version with track changes):*

***Author summary:***

***Why was this study done?***

- *Experimental studies suggest that infection plays a role in neurodegenerative disease development. Supporting evidence in humans is however scarce.*
- *Due to the long preclinical stage of neurodegenerative diseases, it is still unclear whether infection constitutes a risk factor or is merely a comorbidity or secondary event.*
- *No study has explored the association of infections treated in specialized care (i.e., hospital in- and out-patient care) with the subsequent risk of common neurodegenerative diseases (AD, PD, and ALS) in a single population.*

***What did the researchers find?***

- *Infections treated in specialized care were associated with an increased subsequent risk of AD and PD – primarily AD and PD diagnosed before 60 years, but not ALS. These positive associations remained after excluding infections experienced within 10 years before diagnosis of AD or PD.*
- *Increased risks of AD and PD were observed for bacterial, viral, and other infections, and across different sites of infection, including central nervous system, gastrointestinal, and genitourinary infections.*
- *Individuals with repeated infections in early- and mid-life had the greatest risk increment of AD and PD.*

***What do these findings mean?***

- *The underlying mechanisms for the link between infections and neurodegenerative disease are likely not specific to certain pathogens or affected organs but occur at the systemic level.*
- *Infectious events may be a trigger or amplifier of a pre-existing disease process, leading to clinical onset of neurodegenerative disease at a relatively early age among individuals with disease predisposition.*

At line 91 and any other instances, please use the general style "60 years".

**Reply:** We have now edited the manuscript as requested.

"Methods" at line 147.

**Reply:** We have edited this as requested (on page 7).

At the end of the main text, please remove the information on funding from the Acknowledgements (in the event of publication this will appear in the article metadata, via entries in the submission form, and it does not need to be repeated here).

**Reply:** We have removed it from the manuscript as requested.

Please make "p<0.001" the smallest p value quoted throughout, unless there are specific statistical reasons to do otherwise.

**Reply:** We have now edited the manuscript as requested.

Throughout the text, please move reference call-outs prior to punctuation, e.g., "... genetic causes [6,7]." at line 115.

**Reply:** We appreciate your suggestion and have now edited the manuscript thoroughly as requested.

Noting references 4 & 5, for example, please abbreviate journal names consistently. Noting reference 44, please use the journal name abbreviation "PLoS ONE".

**Reply:** Thank you for this suggestion. We have now updated the references as requested.

**Comments from the reviewers:**

***** Reviewer #1:**

The authors have satisfactorily considered and responded to each comment in turn, adding to the analysis and amending the manuscript accordingly.

**Reply:** We appreciate your positive assessment.

***** Reviewer #2:**

The authors have done good job addressing my concerns.

**Reply:** Thank you for the positive assessment.

My few remaining concerns are as follows:

As a response to my question about the time of the recording of the covariates, the authors wrote that "[A]ll covariates were measured on the index date or earlier". I still struggle to understand what this means. Were there covariates for which data were not available up to the index date? If so, for what time period were the data available and used for defining each of these covariates? Please clarify in the manuscript the time periods used for the recording of the covariates.

**Reply:** Thank you for your comment. We have now clarified this in the Methods.

*On page 9:*

*"We identified information on area of residence (3 groups: Northern, Central, and Southern Sweden) from the Total Population Register (information available from 1947 onward) and educational attainment (4 groups: 0-9 years, 10-12 years, ≥13 years, and “missing”) from the Swedish Longitudinal Integrated Database for Health Insurance and Labour Market Studies (information available from 1990 onward) [28]. Family history of neurodegenerative disease (yes/no) was defined as a diagnosis of the disease among first-degree relatives (i.e., biological parents and full siblings) (information available from 1964 onward). We identified parents and full siblings from the Swedish Multi-Generation Register [29]. Charlson comorbidity index (3 groups: 0, 1, and ≥2), as a proxy of general health status, was calculated using data from the National Patient Register (information available from 1964 onward) [30]. All covariates were measured on the index date of the cases and controls."*

In my initial review, I had concerns about missing data for education. I agree with the authors that conducting sensitivity analyses is the best way to address this issue. In the revised manuscript, they have conducted a sensitivity analysis in which they dropped the adjustment for education in the whole sample. This is not wrong, but does not take into account potential confounding from education. Therefore, I think the authors should conduct an additional sensitivity analysis among those with data available for education. Within this group, they should compare an analysis with adjustment for education to an analysis without adjustment for education. I think this would show whether adjustment for education would have any material effect on the results.

**Reply:** We appreciate your suggestion. We have now added a sensitivity analysis among individuals with data available for education and compared the results between a model that adjusted for and a model that did not adjust for education. As expected, we observed very similar results for AD, PD, and ALS between the two models. We have now added this new analysis in Methods and Result.

*Methods, on page 11:*

*"4) Because information on education was available only from 1990 onward, there was a high degree of missingness for this covariate. To assess to what extent this could influence the results, we repeated the main analysis without adjustment for education and additionally compared the results between two models with or without adjustment for education among individuals with data available on education."*

*Result, on page 19:*

*"4) We observed similar results for AD (OR=1.16, 95%CI: 1.15-1.18, P < 0.001), PD (OR=1.04, 95%CI: 1.02-1.06, P < 0.001), and ALS (OR=0.97, 95%CI: 0.92-1.04, P = 0.379) in the model without adjusting for education and when restricting the analysis to individuals with complete data on education, consistent results were also noted between the models with or without adjustment for education (OR=1.10, 95%CI: 1.09-1.12 for AD, P < 0.001; OR=1.03, 95%CI: 1.01-1.05 for PD, P < 0.001; and OR=0.98, 95%CI: 0.92-1.05 for ALS, P = 0.603)."*

I also have a couple of additional points that do not affect the validity of the revised manuscript but would clarify reporting:

Please specify in the methods whether the chosen controls were free from all three assessed neurodegenerative diseases or just the single disease of concern in each analysis (or if some other definition was used).

**Reply:** Thank you for your comment. We have clarified it in Methods.

*On page 8:*

*"Controls should be alive and free of the specific neurodegenerative disease of their matched case when selected."*

p. 17, lines 306-309: The authors write that “Similar results were observed 2) when restricting the analyses to individuals without a family history of the disease, or 3) when defining neurodegenerative diseases through at least two hospital visits concerning the same disease.” Probably I missed it, but I could not find these results. Please report them in the appendix. Please also specify whether you mean that the results were similar to those in the age-stratified analysis described on lines 303-306 on the same page or to those of the main analysis.

**Reply:** Thank you for your comment. As requested, we have now added these results in Result and clarified that the results were similar to those of the main analyses.

*On page 19:*

*"Compared to the main analyses, similar results were observed 2) when restricting the analyses to individuals without a family history of the disease (OR=1.13, 95%CI: 1.12-1.15 for AD, P < 0.001; OR=1.04, 95%CI: 1.02-1.06 for PD, P < 0.001; and OR=0.97, 95%CI: 0.91-1.03 for ALS, P = 0.312) or 3) when defining neurodegenerative diseases through at least two hospital visits concerning the same disease (OR=1.13, 95%CI: 1.11-1.15 for AD, P < 0.001; OR=1.03, 95%CI: 1.02-1.06 for PD, P < 0.001; and OR=0.97, 95%CI: 0.90-1.03 for ALS, P = 0.309)."*

***** Reviewer #3:**

Overall, I am satisfied that the authors have adequately addressed my comments. The new time-stratified analysis is helpful. I also agree with the approach to health seeking behaviour, which could indeed be acting a mediator in this context.

**Reply:** We appreciate your positive assessment.

***** Reviewer #4:**

The revised version of this manuscript has addressed most of my concerns but I remain unconvinced by the approach adopted by the authors. They chose to show unstratified analyses as the main analyses but the results make no sense because of the number of difference as a function of age at exposure, birth-year, age at onset of the neurodegenerative diseases, and sex. The results in the abstract (OR of 1.16 for AD, and 1.04 for PD) are incorrect as they aggregate results over groups with considerable heterogeneity.

My expertise is in AD so I will comment primarily on and early onset AD is a different from late (after age 65) onset AD, the first is primarily genetic and the latter is sporadic. To combine these in the primary results is incorrect. Furthermore, Table S4 (see extract below) shows no association between infection and onset of AD after 60 years, while the association with early onset AD has an OR of 1.93. Combing them yield an OR of 1.16, but is this reasonable?

Given the large sample size, careful analyses of the subgroups could make a contribution to our understanding of the association between infection and neurodegenerative diseases.

| S4 Table. Association between any hospital-treated infection and risk of neurodegenerative disease, stratified analyses by sex, age at diagnosis, calendar period at diagnosis, and year of birth | | | | | | | | | | | |
| --- | --- | --- | --- | --- | --- | --- | --- | --- | --- | --- | --- |
|  | AD | | |  | PD | | |  | ALS | | |
| Group | Infection (case/control) | No infection (case/control) | OR (95% CI) |  | Infection (case/control) | No infection (case/control) | OR (95% CI) |  | Infection (case/control) | No infection (case/control) | OR (95% CI) |
| Sex |  |  |  |  |  |  |  |  |  |  |  |
| Male | 20908/92009 | 115090/587981 | 1.20 (1.18-1.22) |  | 8748/44067 | 48533/242338 | 1.01 (0.98-1.03) |  | 903/4700 | 4870/24165 | 0.95 (0.88-1.04) |
| Female | 28881/133767 | 127062/645948 | 1.13 (1.12-1.15) |  | 8070/37824 | 38568/195366 | 1.07 (1.05-1.11) |  | 890/4484 | 3498/17456 | 1.00 (0.92-1.09) |
| Age at diagnosis |  |  |  |  |  |  |  |  |  |  |  |
| < 60 y | 13196/37418 | 62827/342697 | 1.93 (1.89-1.98) |  | 2110/8600 | 11209/57995 | 1.29 (1.22-1.36) |  | 430/2125 | 1815/9100 | 1.02 (0.90-1.16) |
| ≥ 60 y | 36593/188358 | 179325/891232 | 1.00 (0.98-1.01) |  | 14708/73291 | 75892/379709 | 1.01 (0.99-1.03) |  | 1363/7059 | 6553/32521 | 0.96 (0.90-1.03) |

**Reply:** Thank you for your comments. We agree with the reviewer that results of subgroup analyses could contribute to a better understanding of the association between infection and neurogenerative diseases. In the present study, we indeed conducted several subgroup analyses and found that the associations of hospital-treated infections with the risk of AD and PD varied by age at exposure, birth-year, age at onset of the neurodegenerative diseases, and sex.

We decided to report the unstratified analyses as the main results, because our primary hypothesis was that there was an association between infections and risk of ***any*** AD/PD/ALS and these analyses were *a priori* determined as the primary analyses. Although we did also hypothesize that there might be variation in the associations by age at onset of neurodegenerative disease, etc., this was a secondary hypothesis.

Regardless, we do agree with the reviewer that, given the large difference between the results for relatively early- and late-onset of AD and PD, we should highlight more the results on early-onset AD and PD throughout the manuscript so that it is easier for the readership to get the correct message. We have therefore made the following modifications to the manuscript:

1. In Abstract, we highlighted the results of AD and PD diagnosed before 60 years in "Methods and findings" and "Conclusions".
2. In Author summary, we highlighted the results of AD and PD diagnosed before 60 years.
3. We performed additional analyses for AD and PD diagnosed before 60 years and reported the new analysis in Methods and Result, including a new Table 2.
4. In the Discussion of the main text, we highlighted the differential results by age at diagnosis.

We are certainly willing to reconsider our position if the reviewer and editors would not agree with our strategy.

*Abstract, on page 2:*

*"A hospital-treated infection 5 years ago or earlier was associated with an increased risk of AD (OR=1.16, 95%CI: 1.15-1.18, P < 0.001) and PD (OR=1.04, 95%CI: 1.02-1.06, P < 0.001). The associations were primarily due to AD and PD diagnosed before 60 years (OR=1.93, 95%CI: 1.89-1.98 for AD, P < 0.001; OR=1.29, 95%CI: 1.22-1.36 for PD, P < 0.001). "*

*Abstract, on page 3:*

*"Hospital-treated infections, especially in early- and mid-life, were associated with an increased risk of AD and PD, especially of AD and primarily among cases diagnosed at relatively young age."*

*Author summary, on page 4:*

*"* ***What did the researchers find?***

- *Infections treated in specialized care were associated with an increased subsequent risk of AD and PD – primarily AD and PD diagnosed before 60 years, but not ALS. These positive associations remained after excluding infections experienced within 10 years before diagnosis of AD or PD."*

*Methods, on page 10:*

*"Because the positive associations were mainly noted for AD and PD diagnosed before 60 years, and the etiologies for AD and PD diagnosed at relatively young age are potentially different from those diagnosed at later age, we repeated all analyses for AD and PD diagnosed before 60 years specifically."*

*Result, on pages 17-19:*

*"In the separate analyses for AD and PD diagnosed before 60 years, we found largely similar result pattern but with stronger associations, compared with the analysis on any AD and PD (Table 2)."*

| Table 2. Association between hospital-treated infection and risk of AD or PD diagnosed before 60 years. | | | |
| --- | --- | --- | --- |
|  | OR (95% CI) | | |
| Group | AD |  | PD |
| Analysis by infection type |  |  |  |
| Bacterial | 2.08 (2.02-2.15) |  | 1.30 (1.21-1.39) |
| Viral | 1.87 (1.79-1.94) |  | 1.36 (1.24-1.49) |
| Others | 1.85 (1.73-1.98) |  | 1.33 (1.16-1.53) |
| Analysis by infection site |  |  |  |
| CNS | 1.63 (1.49-1.78) |  | 1.39 (1.15-1.69) |
| Gastrointestinal | 1.35 (1.28-1.41) |  | 1.18 (1.06-1.32) |
| Respiratory | 1.63 (1.57-1.70) |  | 1.33 (1.22-1.46) |
| Genitourinary | 2.75 (2.54-2.98) |  | 1.26 (1.04-1.52) |
| Skin | 1.89 (1.77-2.02) |  | 1.16 (0.99-1.36) |
| Analysis by age at infection |  |  |  |
| <40 y | 1.95 (1.90-2.00) |  | 1.36 (1.28-1.45) |
| 40-59.9 y | 1.72 (1.62-1.82) |  | 1.08 (0.99-1.18) |
| Analysis by age and frequency of infection |  |  |  |
| <40 y |  |  |  |
| 0 | Ref. |  | Ref. |
| 1 | 1.73 (1.69-1.78) |  | 1.27 (1.18-1.37) |
| ≥ 2 | 2.70 (2.59-2.82) |  | 1.63 (1.47-1.82) |
| 40-59.9 y |  |  |  |
| 0 | Ref. |  | Ref. |
| 1 | 1.64 (1.53-1.76) |  | 1.02 (0.92-1.14) |
| ≥ 2 | 1.97 (1.76-2.20) |  | 1.21 (1.05-1.41) |
| Analysis by sex |  |  |  |
| Male | 1.76 (1.70-1.81) |  | 1.17 (1.08-1.27) |
| Female | 2.18 (2.11-2.26) |  | 1.42 (1.31-1.54) |
| Analysis by calendar period at diagnosis |  |  |  |
| 1970-1986 | 2.39 (2.26-2.52) |  | 1.42 (1.25-1.62) |
| 1987-2000 | 1.88 (1.83-1.93) |  | 1.59 (1.45-1.75) |
| 2001-2016 | 1.35 (1.20-1.52) |  | 1.04 (0.95-1.13) |
| Analysis by year of birth |  |  |  |
| 1900-1919 | 2.08 (1.35-3.19) |  | 2.59 (1.49-4.48) |
| 1920-1939 | 1.87 (1.75-1.99) |  | 1.30 (1.14-1.48) |
| 1940-1959 | 2.15 (2.08-2.23) |  | 1.14 (1.05-1.23) |
| ≥ 1960 | 1.59 (1.52-1.65) |  | 1.39 (1.24-1.56) |
| CI, confidence interval; OR, odds ratio. Conditional on matching factors (age and sex) and further adjusted for area of residence, educational attainment, family history of the disease, and history of comorbidity. Infections diagnosed during five years before the index date were excluded to alleviate the potential influence of reverse causation due to diagnostic delay. | | | |
| *P*<0.001 for all associations, except for PD in the strata of skin (*P*=0.071), 40-59.9 y (P=0.093), 40-59.9 y with one infection (*P*=0.692), and 2001-2016 (*P*=0.386). | | | |

*Discussion, on page 20:*

*"In this nationwide study, we found that hospital-treated infections 5 years ago or earlier were associated with an increased risk of AD and PD, especially of AD and primarily among cases diagnosed before 60 years, but not ALS."*

*Discussion, on pages 20-21:*

*"Our finding that hospital-treated infections were more strongly associated with risk of AD and PD before 60 years, compared to later, and that individuals with repeated infections in early- and mid-life had the greatest risk increment of AD and PD, are new and potentially important."*

*Discussion, on page 26:*

*“In conclusion, our study suggested that individuals with hospital-treated infections, especially in early- and mid-life, had an increased risk of developing AD and PD, especially of AD and primarily for cases diagnosed at young age. Further studies are warranted to validate these findings, to elucidate underlying mechanisms, and to determine whether better control of hospital-treated infections could prevent or delay onset of neurodegenerative diseases, especially the ones with an onset relatively early in life.”*
