# Supplementary material for: Hospital-treated infections in early- and mid-life and risk of Alzheimer’s disease, Parkinson’s disease, and amyotrophic lateral sclerosis: A nationwide nested case-control study in Sweden
Source: PLoS Med. 2022 Sep 15;19(9):e1004092. doi: 10.1371/journal.pmed.1004092 (PMC9477309; doi:10.1371/journal.pmed.1004092)
Supplement: Attachment — Submitted filename: letter to editor and reviewers_R3.docx [file pmed.1004092.s015.docx]

**Requests from Editors:**

**From the academic editor:**

The association is primarily being driven by the early-onset cases (<60 y). Once these are excluded, the OR is close to 1.0. Moreover, Fig 4 shows that 2 or more infections after 60 were in fact protective for AD ((OR 0.91 (0.89-0.93)), so to speak. It is true that the authors should look at the whole group first, but when they try to understand this deeper, the finding of early-onset stands out. This should be highlighted, and it is intriguing and counter-intuitive. The authors appear to de-emphasise this finding in favour of the whole group, but the message from the analysis appears to be that the finding is true only for early-onset cases.

**Reply:**

Thank you for your comments. We have now further highlighted the results on cases diagnosed before 60 years throughout the manuscript so that it is easier for the readership to get the correct message. We have made the following modifications to the manuscript:

- In Abstract, we highlight the results of AD and PD diagnosed before 60 years in "Methods and findings" and "Conclusions";
- In Author summary, we highlight the results of AD and PD diagnosed before 60 years;
- In Methods and Results of the main text, we repeat all main analyses for AD and PD diagnosed before 60 years and report the results in Table 3;
- In Discussion of the main text, we highlight the differential results by age at diagnosis.

Abstract, on page 3 (page number in the tracked version):

*"* *The associations were primarily due to AD and PD diagnosed before 60 years (OR=1.93, 95%CI: 1.89-1.98 for AD, P < 0.001; OR=1.29, 95%CI: 1.22-1.36 for PD, P < 0.001), whereas no association was found for those diagnosed at 60 years or older (OR=1.00, 95%CI: 0.98-1.01 for AD, P = 0.508; OR=1.01, 95%CI: 0.99-1.03 for PD, P = 0.382)."*

Abstract, on page 3:

*"Hospital-treated infections, especially in early- and mid-life, were associated with an increased risk of AD and PD, primarily among AD and PD cases diagnosed before 60 years."*

Author summary, on page 4:

*"Infections treated in specialized care were associated with an increased subsequent risk of AD and PD – primarily AD and PD diagnosed before 60 years, but not ALS."*

Methods, on page 11:

*"* *Because the positive associations were mainly noted for those diagnosed before 60 years, and the etiologies for AD and PD diagnosed at relatively young age are potentially different from those diagnosed at later age, we repeated all analyses for AD, PD, and ALS diagnosed before or after 60 years, separately."*

Results, on page 18:

*"All these results were primarily attributable to AD and PD diagnosed before 60 years (Table 3), compared with those diagnosed at 60 years or older (S6 Table). For instance, genitourinary infection was associated with the highest risk of AD before 60 years (OR=2.75, 95%CI: 2.54-2.98, P < 0.001), whereas CNS infection was associated the highest risk of PD before 60 years (OR=1.39, 95%CI: 1.15-1.69, P < 0.001). Gastrointestinal infection was also associated with a higher risk of AD (OR=1.35, 95%CI: 1.28-1.41, P < 0.001) and PD (OR=1.18, 95%CI: 1.06-1.32, P < 0.001) diagnosed before 60 years."*

Discussion, on page 23:

*"In this nationwide study, we found that hospital-treated infections 5 years ago or earlier were associated with an increased risk of AD and PD, specifically among cases diagnosed before 60 years, but not ALS."*

Discussion, on page 24:

*"Although unexpected, our subsequent findings that hospital-treated infections were more strongly associated with risk of AD and PD before 60 years, compared to later, and that individuals with repeated infections in early- and mid-life had the greatest risk increment of AD and PD, are new and potentially important."*

Discussion, on page 29:

*"In conclusion, our study suggested that individuals with hospital-treated infections, especially in those occurring in early- and mid-life, had an increased risk of developing AD and PD, attributable to cases diagnosed before 60 years."*

**Other editorial points:**

2. Response to reviewers: Please address the remaining comments of Reviewer 4. Specifically, please discuss and present a figure/table of results broken down by age of diagnosis (i.e. older or younger than 60 years of age) for AD/PD/ ALS (sex, age at infection, type of infection, calendar period). Please also address the remaining issues raised by Reviewer 2.

**Reply:** The analyses suggested by Reviewer 4 and the remaining issues raised by Reviewer 2 have now been addressed in the manuscript. Please see our detailed responses below.

3. Title: Please revise as follows and please capitalize the first word of the subtitle and please make this change in the manuscript submission system as well as the manuscript file. “Hospital-treated infections in early- and mid-life and risk of Alzheimer’s disease, Parkinson’s disease, and amyotrophic lateral sclerosis: A nationwide nested case-control study in Sweden”

**Reply:** We have revised the title as suggested.

4. Competing interests: Please revise to “independent of the present study” in your statement.

**Reply:** We have edited this as requested.

5. Abstract: Line 33: Please mention the variables that are adjusted for in the analyses.

**Reply:** We have added such information as requested.

Abstract, on page 2:

*"Conditional logistic regression was used to estimate odds ratios (ORs) and 95% confidence intervals (CIs) with adjustment for potential confounders, including sex, year of birth, area of residence, educational attainment, family history of neurodegenerative disease, and Charlson comorbidity index."*

6. Abstract: Line 36-37: Please revise to: “A hospital-treated infection 5 or more years earlier was associated with…”

**Reply:** We have revised this sentence as requested (on page 2).

7. Abstract: Line 38-39: For the sake of comparison, please also present the results for those diagnosed at age 60 years or older. “The associations were primarily due to AD and PD diagnosed before 60 years”

**Reply:** We have presented the results for those diagnosed at age 60 years or older as requested.

Abstract, on page 3:

*“The associations were primarily due to AD and PD diagnosed before 60 years (OR=1.93, 95%CI: 1.89-1.98 for AD, P < 0.001; OR=1.29, 95%CI: 1.22-1.36 for PD, P < 0.001), whereas no association was found for those diagnosed at age 60 years or older (OR=1.00, 95%CI: 0.98-1.01 for AD, P = 0.508; OR=1.01, 95%CI: 0.99-1.03 for PD, P = 0.382)."*

8. Abstract: Line 44: Please delete “however” from the sentence.

**Reply:** We have deleted this word from the sentence (on page 3).

9. Abstract: Line 51: We suggest “especially of AD” is not needed here as the association was apparent for both AD and PD.

**Reply:** We have revised the sentence.

Abstract, on page 3:

*"Hospital-treated infections, especially in early- and mid-life, were associated with an increased risk of AD and PD, primarily among AD and PD cases diagnosed before 60 years."*

10. Author summary: Line 89-90: We suggest revising to: “The underlying mechanisms for the link between infections and neurodegenerative disease may not be specific to certain pathogens or affected organs but possibly occur at the systemic level.”

**Reply:** We have edited this as requested (on page 5).

11. Methods: Lines 164-168: Rather than referring to sensitivity/PPV as “varying” please comment on the relatively lower sensitivity/PPV in particular for AD and why this might be the case: “The register-based definitions of AD, PD, and ALS have been validated against gold-standard clinical workup, showing a high specificity but a varying sensitivity and positive predictive value for AD (99.7%, 32.5%, and 57%) [23], PD (>98%, 72%, and 71%) [24], and ALS (all >90%) [25]. Date of diagnosis was defined as the date of first hospital visit concerning the disease.

**Reply:** We appreciate your suggestion and have now discussed this in Methods and Discussion.

Methods, on page 8:

*“The relatively low sensitivity of AD diagnosis is likely attributable to misdiagnosis of AD as other dementias [23].”*

Discussion, on page 28:

*"In addition, as the sensitivity of register-based definition is low for AD (32.5%) [23], although higher for PD (72%) [24] and ALS (>90%) [25], not all patients with neurodegenerative diseases were identified, which might have diluted the real associations toward null."*

12. Methods: Lines 167-168: Please clarify if the date of diagnosis was the first date where AD/PD/ALS was recorded as the primary diagnosis in the register.

**Reply:** Thank you for your comment. We have now clarified it in Methods.

Methods, on page 8:

*"Date of diagnosis was defined as the date of first hospital visit concerning the disease as either the primary or a secondary diagnosis."*

13. Methods: Lines 175-176: Thank you for including a copy of the pre-specified analysis plan as a supporting information file. In the analysis plan, you note that updates were made to the plan in response to peer review. Please make sure that any changes in the analysis-- including those made in response to peer review comments-- are identified as such in the Methods section of the paper, with rationale.

**Reply:** We confirm that all changes can be identified as such in Methods and with rationale.

14. Results: Lines 275-277: Please reference the figure/table where these results are presented: “Positive associations for AD and PD were similarly observed for bacterial, viral, and other infections, as well as for CNS, gastrointestinal, and genitourinary infections.”

**Reply:** We have added the information as requested.

Results, on page 15:

*"Positive associations for AD and PD were similarly observed for bacterial, viral, and other infections, as well as for CNS, gastrointestinal, and genitourinary infections (Table 2)."*

15. Results: Lines 290-291: “Stronger associations were also observed for AD ascertained before 65, compared with AD ascertained after 65” Please present the OR, 95% CI and p values in the text for this finding.

**Reply:** We have added the information as requested.

Results, on page 17:

*"Stronger associations were also observed for AD ascertained before 65 (e.g., for individuals with ≥2 events of infections before 40 years, OR=1.95, 95%CI: 1.91-2.00, P < 0.001), compared with AD ascertained after 65 (OR=1.11, 95%CI: 1.05-1.18, P < 0.001) (S5 Table)."*

16. Results: Line 299-302: Please mention the table/figure where the analysis with number of infections is presented.

**Reply:** We have added the information as requested.

Results, on page 17:

*"Dose-response relationships were observed for frequency of infections before 40 years (all P _for trend_ < 0.001). For example, individuals with ≥2 events of infections before 40 years had the highest risk of AD (OR=2.62, 95%CI: 2.52-2.72, P < 0.001) and PD (OR=1.41, 95%CI: 1.29-1.53, P < 0.001) (S3 Table)."*

17. Results: Line 312-313: “Gastrointestinal infection was also associated with a higher risk of AD (OR=1.35, 95%CI: 1.28-1.41, P < 0.001) and PD (OR=1.18, 95%CI: 1.06-1.32, P < 0.001).” Please clarify if this is among all individuals or those diagnosed before age 60 years.

**Reply:** Thank you for your comment. We have clarified it in Results.

Results, on page 18:

*"Gastrointestinal infection was also associated with a higher risk of AD (OR=1.35, 95%CI: 1.28-1.41, P < 0.001) and PD (OR=1.18, 95%CI: 1.06-1.32, P < 0.001) diagnosed before 60 years."*

18. Results: Lines 321-346: In the description of secondary analyses, we suggest removing the numbering from the paragraph (“1) A stronger association…2) when restricting the analyses…).

**Reply:** We have removed the numbering as requested.

19. Results: Lines 325-339: Please include supporting information tables presenting the results of analyses restricted to those without a family history, neurodegenerative diseases defined by at least 2 hospital visits, from models not adjusted for education or restricted to those with complete education data, and when those with more than one neurodegenerative disease were excluded.

**Reply:** We have now added such information in a new S7 Table (**also see Table 1 below**).

| Table 1. Sensitivity analyses for the association between any hospital-treated infection and risk of neurodegenerative disease (5-year lag time) | | | | | | | | | | | | | | |
| --- | --- | --- | --- | --- | --- | --- | --- | --- | --- | --- | --- | --- | --- | --- |
|  | AD | | | |  | PD | | | |  | ALS | | | |
| Sensitivity analysis | Infection (case/control) | No infection (case/control) | OR (95% CI) | *P* |  | Infection (case/control) | No infection (case/control) | OR (95% CI) | *P* |  | Infection (case/control) | No infection (case/control) | OR (95% CI) | *P* |
| Without a family history of the disease | 45549/217164 | 228047/1188019 | 1.13 (1.12-1.15) | <0.001 |  | 16240/80235 | 85302/432272 | 1.04 (1.02-1.06) | <0.001 |  | 1748/9159 | 8232/41520 | 0.97 (0.91-1.03) | 0.312 |
| At least two hospital visits about the same disease | 26629/124081 | 126908/643604 | 1.13 (1.11-1.15) | <0.001 |  | 11407/58676 | 58736/292039 | 1.03 (1.02-1.06) | <0.001 |  | 1398/7208 | 6087/30217 | 0.97 (0.91-1.04) | 0.354 |
| In whole population, without adjust for education | 49789/225776 | 242152/1233929 | 1.16 (1.15-1.18) | <0.001 |  | 16818/81891 | 87101/437704 | 1.04 (1.02-1.06) | <0.001 |  | 1793/9184 | 8368/41621 | 0.97 (0.92-1.03) | 0.379 |
| In those with data available for education, adjust for education | 32598/151528 | 107303/547548 | 1.103 (1.087-1.118) | <0.001 |  | 12849/63690 | 45211/225638 | 1.034 (1.012-1.054) | 0.012 |  | 1602/8096 | 5438/26935 | 0.984 (0.923-1.049) | 0.613 |
| In those with data available for education, not adjust for education | 32598/151528 | 107303/547548 | 1.103 (1.088-1.119) | <0.001 |  | 12849/63690 | 45211/225638 | 1.033 (1.011-1.052) | 0.017 |  | 1602/8096 | 5438/26935 | 0.983 (0.922-1.048) | 0.602 |
| After excluding individuals with multiple neurodegenerative diseases | 47802/216421 | 229712/1171149 | 1.18 (1.16-1.19) | <0.001 |  | 15273/74692 | 76595/384648 | 1.04 (1.02-1.06) | <0.001 |  | 1696/8801 | 8020/39779 | 0.97 (0.91-1.03) | 0.323 |
| AD: Alzheimer’s disease; ALS: amyotrophic lateral sclerosis; CI, confidence interval; OR, odds ratio; PD: Parkinson’s disease. Conditional on matching factors (sex and year of birth) and further adjusted for area of residence, educational attainment, family history of the disease, and history of comorbidity. Infections diagnosed during five years before the index date were excluded to alleviate the potential influence of reverse causation due to diagnostic delay and surveillance bias. | | | | | | | | | | | | | | |

20. Results: As suggested by reviewer 4, please also present a table of results broken down by age of diagnosis (i.e. older or younger than 60 years of age) for AD/PD/ALS (sex, age at infection, type of infection, calendar period).

**Reply:** We have now added these results as Table 3 in the main text and S6 Table in supplementary materials. We have updated the Methods and Results of the main text accordingly.

Methods, on page 11:

*"* *Because the positive associations were mainly noted for those diagnosed before 60 years, and the etiologies for AD and PD diagnosed at relatively young age are potentially different from those diagnosed at later age, we repeated all analyses for AD, PD, and ALS diagnosed before or after 60 years, separately."*

Results, on page 18:

*"All these results were primarily attributable to AD and PD diagnosed before 60 years (Table 3), compared with those diagnosed at 60 years or older (S6 Table). For instance, genitourinary infection was associated with the highest risk of AD before 60 years (OR=2.75, 95%CI: 2.54-2.98, P < 0.001), whereas CNS infection was associated the highest risk of PD before 60 years (OR=1.39, 95%CI: 1.15-1.69, P < 0.001). Gastrointestinal infection was also associated with a higher risk of AD (OR=1.35, 95%CI: 1.28-1.41, P < 0.001) and PD (OR=1.18, 95%CI: 1.06-1.32, P < 0.001) diagnosed before 60 years."*

21. Discussion: Line 349-350, Please revise to: “In this nationwide study, we found that hospital-treated infections 5 years ago or earlier were associated with an increased risk of AD and PD, specifically among cases diagnosed before 60 years, but not ALS.”

**Reply:** We have revised the sentence as requested (on page 23).

22. Discussion: Line 355-357: Please revise to: “Though causality cannot be inferred from the study, the similar results for different infection types and sites might suggest that the potential underlying mechanisms of the observed associations are not specific to certain pathogens, and raise the possibility that systemic inflammation might play a role in brain health, an idea that is supported by evidence from a previous study reporting a relationship between hospital treated infection and vascular dementia and AD [17].” or similar.

**Reply:** We appreciate your suggestion and have edited the sentence as requested.

Discussion, on page 24:

*"Though causality cannot be inferred from the study, the similar results for different infection types and sites might suggest that the potential underlying mechanisms of the observed associations are not specific to certain pathogens, and raise the possibility that systemic inflammation might play a role in brain health, an idea that is supported by evidence from a previous study reporting a relationship between hospital-treated infection and vascular dementia and AD [17]."*

23. Discussion: Line 385-387: Please revise this statement, as the paper cited actually reports finding no significant association between risk of PD more than 10 years after infection: “A recent study from Denmark reported an increased risk of PD more than 10 years after exposure to infections (OR=1.04, 95%CI: 0.98-1.10) [12].”

**Reply:** We have edited the sentence as suggested.

Discussion, on page 25:

*"A recent study from Denmark reported an increased risk of PD with similar magnitude as the present study more than 10 years after exposure to infections, however the result was not statistically significant (OR=1.04, 95%CI: 0.98-1.10) [12]."*

24. Discussion: Line 392-393: Please clarify if this refers to calendar year of diagnosis: “...the magnitude of the associations decreased over time.” Please comment on how the attenuation of the association in more recent calendar years might arise from better treatment for infection or improved diagnosis for AD/PD.

**Reply:** We have now clarified this and commented on how better treatment for infection and improved diagnosis for AD/PD might affect the results.

Discussion, on pages 25-26

*"In the present study, although positive associations were observed for AD and PD diagnosed before 60 years and for infections in early- and mid-life throughout the study period, the magnitude of the associations decreased over calendar period. There are likely multiple explanations. First, the treatment of infections might have changed during the study period, with presumably better treatment outcomes during later calendar periods than before. Similarly, the diagnosis of neurodegenerative diseases might have improved over calendar period, not only thanks to improvements in diagnostic tools, especially imaging and more recently also soluble biomarkers, but also due to improved societal awareness of these diseases and resources in neurology and geriatric care."*

25. Discussion: Line 458-459: Please provide a reference: “The low sensitivity of AD diagnosis was likely attributable to misdiagnosis of AD as other dementias.”

**Reply:** We have now relocated this sentence into the "Methods" and added the reference.

Methods, on page 8:

*“The relatively low sensitivity of AD diagnosis is likely attributable to misdiagnosis of AD as other dementias [23].”*

26. Discussion: Line 470-473: Please comment on how the relatively young study population might impact the generalizability and interpretation of the findings, in light of the fact that associations were seen only among those diagnosed younger than 60 years of age.

**Reply:** We have now commented this in Discussion.

Discussion, on page 29:

*"Moreover, due to varying incidence of infections and neurodegenerative diseases across countries [2,3,8] and relatively young participants of the present study, generalization of our findings to other settings should be done with caution. Similarly, whether hospital-treated infections indeed do not influence the risk of AD and PD beyond 60 years of age needs to be studied further."*

27. Discussion: Line 478: Please use “substance misuse” here.

**Reply:** We have edited it as requested (on page 29).

28. Discussion: Line 485-487: Please revise to: “In conclusion, our study suggested that individuals with hospital-treated infections, especially in those occurring in early- and mid-life, had an increased risk of developing AD and PD, attributable to cases diagnosed at young age.”

**Reply:** We have revised the sentence as requested.

Discussion, on page 29:

*"In conclusion, our study suggested that individuals with hospital-treated infections, especially in those occurring in early- and mid-life, had an increased risk of developing AD and PD, attributable to cases diagnosed before 60 years."*

29. References: The journal abbreviation for reference 44 should be PLoS One.

**Reply:** We have now updated the reference as requested.

30. STROBE checklist: For item 22, please refer to the location as “Funding” or similar.

**Reply:** We have now updated the item 22 as requested.

31. Figure 1: Please define all abbreviations (AD/PD/ALS) in the legend.

**Reply:** We have now defined all abbreviations in the legend.

On page 15:

*"Fig 1. Percentage of individuals with hospital-treated infections among patients with neurodegenerative diseases and their matched controls during the 20 years before the index date. AD: Alzheimer’s disease; ALS: amyotrophic lateral sclerosis; PD: Parkinson’s disease."*

32. Figure 2, Figure 3, Figure 4: Please define all abbreviations in the legend. Please add numbers of cases/controls and those with and without infection, as well as p values to the figure. Please note the factors adjusted for in the legends. Please also provide the results of unadjusted analyses (these can be added to the supporting information if preferred).

**Reply:** To incorporate the information as suggested, we have now replaced the 3 figures with 3 tables (i.e., Fig 2 as Table 2, Fig 3 as S4 Table, and Fig 4 as S3 Table).

Because of the individually matched design of the nested case-control study, all results are inherently adjusted for matching factors (sex and year of birth). We can therefore not present completely unadjusted results. We are certainly willing to reconsider our position if the editor does not agree with us.

33. Table 2: Please add p values to the table. In the legend, please define all abbreviations used (AD/PD/CNS).

**Reply:** We have now modified the table accordingly (named as Table 3 in the revised manuscript).

34. S3 -S10 Table: Please include p values. Please define AD, PD, ALS in the legends. Please also provide the unadjusted comparisons as well as the adjusted comparisons where applicable.

**Reply:** Thank you for the suggestion. We have now added p values and defined all abbreviations in the supplementary tables. We choose not to present the results of unadjusted analyses – please see our response to the comment above.

**Comments from Reviewers:**

**Reviewer #1:**

The authors have satisfactorily considered and responded to each comment in turn, adding to the analysis and amending the manuscript accordingly.

**Reply:** We appreciate your positive feedback.

**Reviewer #2:**

The authors have responded to all my concerns, but their response needs to be clarified. When reporting the results of the sensitivity analyses suggested by me, they say that (p. 19, lines 333-336) "when restricting the analysis to individuals with complete data on education, consistent results were also noted between the models with or without adjustment for education (OR=1.10, 95%CI: 1.09- 1.12 for AD, P < 0.001; OR=1.03, 95%CI: 1.01-1.05 for PD, P < 0.001; and OR=0.98, 95%CI: 0.92-1.05 for ALS, P = 0.603)." Here, to support their text, they should show two ORs for each of the diseases (AD, PD, and ALS), one for the model adjusted for education and the other for the model not adjusted for education.

**Reply:** We appreciate your comment. The magnitude of OR and its 95%CI between the models with or without adjustment for education is identical (at a precision level of 2 decimals). We have now added the results in S7 Table and revised the sentence to clarify.

Results, on pages 22-23:

*"We observed similar results in the model without adjusting for education and when restricting the analysis to individuals with complete data on education, consistent results were also noted between the models with or without adjustment for education (S7 Table)."*

While reading the paper, I also noticed another point about clarifying the Discussion: On p. 21 (lines 372-376) the authors refer to an earlier study saying that "Another study reported a dose-response relationship between number of infectious diseases and dementia risk [17]. This study however did not explore the effect of age at infection and whether a dose-response relationship existed still after considering the influence of potential reverse causation due to the pre-clinical stage and diagnostic delay of neurodegenerative diseases." In a previous version of the manuscript they specified that the previous study did not consider the influence of potential reverse causation when looking at the dose-response relationship between dementia and AD, which was true. Now that the mention of AD has been omitted, the statement is not any more accurate as there was such an analysis on all-cause dementia in the previous study.

**Reply:** We appreciate your comment and have now modified the sentence to make it more accurate.

*Discussion, on pages 24-25:*

*"This study however did not explore the effect of age at infection and whether a dose-response relationship existed still for AD after considering the influence of potential reverse causation due to the pre-clinical stage and diagnostic delay of AD."*

**Reviewer #4:**

Hospital-treated infections in early- and mid-life and risk of Alzheimer's disease, Parkinson's disease, and amyotrophic lateral sclerosis: a nationwide nested case-control study in Sweden" (PMEDICINE-D-22-01054R3)

1.Apologies for repeating what I said earlier but the revised version of the manuscript remains problematic. The unstratified analyses make no sense because of the number of difference as a function of age at exposure, birth-year, age at onset of the neurodegenerative diseases, and sex. The results section of the abstract provides results that are incorrect, see below: "A hospital-treated infection 5 years ago or earlier was associated with an increased risk of AD (OR=1.16, 95%CI: 1.15-1.18, P < 0.001) and PD (OR=1.04, 95%CI: 1.02-1.06, P < 0.001)."

**Reply:**

Thank you for your comments. We have now further highlighted the results on cases diagnosed before 60 years throughout the manuscript so that it is easier for the readership to get the correct message. We have made the following modifications to the manuscript:

- In Abstract, we highlight the results of AD and PD diagnosed before 60 years in "Methods and findings" and "Conclusions";
- In Author summary, we highlight the results of AD and PD diagnosed before 60 years;
- In Methods and Results of the main text, we repeat all main analyses for AD and PD diagnosed before 60 years and report the results in Table 3;
- In Discussion of the main text, we highlight the differential results by age at diagnosis.

Abstract, on page 3 (page number in the tracked version):

*"* *The associations were primarily due to AD and PD diagnosed before 60 years (OR=1.93, 95%CI: 1.89-1.98 for AD, P < 0.001; OR=1.29, 95%CI: 1.22-1.36 for PD, P < 0.001), whereas no association was found for those diagnosed at 60 years or older (OR=1.00, 95%CI: 0.98-1.01 for AD, P = 0.508; OR=1.01, 95%CI: 0.99-1.03 for PD, P = 0.382)."*

Abstract, on page 3:

*"Hospital-treated infections, especially in early- and mid-life, were associated with an increased risk of AD and PD, primarily among AD and PD cases diagnosed before 60 years."*

Author summary, on page 4:

*"Infections treated in specialized care were associated with an increased subsequent risk of AD and PD – primarily AD and PD diagnosed before 60 years, but not ALS."*

Methods, on page 11:

*"* *Because the positive associations were mainly noted for those diagnosed before 60 years, and the etiologies for AD and PD diagnosed at relatively young age are potentially different from those diagnosed at later age, we repeated all analyses for AD, PD, and ALS diagnosed before or after 60 years, separately."*

Results, on page 18:

*"All these results were primarily attributable to AD and PD diagnosed before 60 years (Table 3), compared with those diagnosed at 60 years or older (S6 Table). For instance, genitourinary infection was associated with the highest risk of AD before 60 years (OR=2.75, 95%CI: 2.54-2.98, P < 0.001), whereas CNS infection was associated the highest risk of PD before 60 years (OR=1.39, 95%CI: 1.15-1.69, P < 0.001). Gastrointestinal infection was also associated with a higher risk of AD (OR=1.35, 95%CI: 1.28-1.41, P < 0.001) and PD (OR=1.18, 95%CI: 1.06-1.32, P < 0.001) diagnosed before 60 years."*

Discussion, on page 23:

*"In this nationwide study, we found that hospital-treated infections 5 years ago or earlier were associated with an increased risk of AD and PD, specifically among cases diagnosed before 60 years, but not ALS."*

Discussion, on page 24:

*"Although unexpected, our subsequent findings that hospital-treated infections were more strongly associated with risk of AD and PD before 60 years, compared to later, and that individuals with repeated infections in early- and mid-life had the greatest risk increment of AD and PD, are new and potentially important."*

Discussion, on page 29:

*"In conclusion, our study suggested that individuals with hospital-treated infections, especially in those occurring in early- and mid-life, had an increased risk of developing AD and PD, attributable to cases diagnosed before 60 years."*

2.This is also the case in the following sections of the authors summary; these statements suggest that infection is a risk factor for AD. Early onset AD is different from AD and the distinction is lost in these sentences.

***What did the researchers find?***

*Individuals with repeated infections in early- and mid-life had the greatest risk increment of AD and PD.*

***What do these findings mean?***

*The underlying mechanisms for the link between infections and neurodegenerative disease are likely not specific to certain pathogens or affected organs but occur at the systemic level.*

*Infectious events may be a trigger or amplifier of a pre-existing disease process, leading to clinical onset of neurodegenerative disease at a relatively early age among individuals with disease predisposition.*

**Reply:** We appreciate your comment and have now highlighted the findings on early-onset AD.

On pages 4-5:

*"What did the researchers find?*

- ***Infections treated in specialized care were associated with an increased subsequent risk of AD and PD – primarily AD and PD diagnosed before 60 years, but not ALS.*** *These positive associations remained after excluding infections experienced within 10 years before diagnosis of AD or PD.*
- *Increased risks of AD and PD were observed for bacterial, viral, and other infections, and across different sites of infection, including central nervous system, gastrointestinal, and genitourinary infections.*
- *Individuals with repeated infections in early- and mid-life had the greatest risk increment of AD and PD.*

*What do these findings mean?*

- *The underlying mechanisms for the link between infections and neurodegenerative disease may not be specific to certain pathogens or affected organs but possibly occur at the systemic level.*
- *Infectious events may be a trigger or amplifier of a pre-existing disease process, leading to clinical onset of neurodegenerative disease* ***at a relatively early age*** *among individuals with disease predisposition. "*

3.In the response to reviewers comments the authors state the following:

"We decided to report the unstratified analyses as the main results, because our primary hypothesis was that there was an association between infections and risk of any AD/PD/ALS and these analyses were a priori determined as the primary analyses. Although we did also hypothesize that there might be variation in the associations by age at onset of neurodegenerative disease, etc., this was a secondary hypothesis."

If this is the case then the authors ought to state clearly that their primary hypothesis was not verified and subsequent analyses showed an association for early onset AD/PD.

**Reply:** We agree and have now clarified this in Discussion.

Discussion, on page 24:

*"Although unexpected, our subsequent findings that hospital-treated infections were more strongly associated with risk of AD and PD before 60 years, compared to later, and that individuals with repeated infections in early- and mid-life had the greatest risk increment of AD and PD, are new and potentially important."*

4.Following on from the previous comment, I don't quite understand the analyses reported in Figure 4. Surely, this ought to be stratified by age at onset of AD/PD.

**Reply:** We have now removed Figure 4, and reported the results for cases diagnosed <60 and ≥60 years in Table 3 and S6 Table, respectively.
